# Adjunctive Methylprednisolone After Thrombectomy: A Secondary Analysis Stratified by Admission White Blood Cell Count

**DOI:** 10.1002/cns.70956

**Published:** 2026-05-31

**Authors:** Chengsong Yue, Jie Yang, Dahong Yang, Guanting Heng, Linyu Li, Shihai Yang, Xuanyu Chen, Yi Lin, Kunxin Lin, Wenjie Zi

**Affiliations:** ^1^ Department of Neurology, Xinqiao Hospital and the Second Affiliated Hospital Army Medical University (Third Military Medical University) Chongqing China; ^2^ Department of Neurology The First Affiliated Hospital of Fujian Medical University Fuzhou China; ^3^ Department of Neurology The Second Affiliated Hospital of Chongqing Medical University Chongqing China

**Keywords:** blood–brain barrier, endovascular thrombectomy, inflammation, leukocytes, methylprednisolone, stroke, white blood cell count

## Abstract

**Background:**

Leukocyte‐driven inflammation may contribute to reperfusion–outcome mismatch after thrombectomy. We tested whether admission white blood cell (WBC) count may identify reperfused patients more likely to benefit from adjunctive methylprednisolone.

**Methods:**

This post hoc secondary analysis of the MARVEL randomized, placebo‐controlled trial was conducted with a prospectively finalized statistical analysis plan. We included patients with anterior‐circulation large‐vessel occlusion stroke who achieved successful reperfusion after thrombectomy and received intravenous methylprednisolone or placebo. The analysis included 1201 patients stratified by admission WBC count (< 10 × 10^9^/L, *n* = 808; ≥ 10 × 10^9^/L, *n* = 393). The primary outcome was 90‐day modified Rankin Scale (mRS 0–6) using covariate‐adjusted ordinal logistic regression. Secondary outcomes included mRS 0–3 and 0–4, early NIHSS, mortality, symptomatic intracranial hemorrhage (sICH), any intracranial hemorrhage, pneumonia, and gastrointestinal bleeding.

**Results:**

The treatment × WBC interaction reached nominal statistical significance (*p* = 0.04). In patients with WBC ≥ 10 × 10^9^/L, methylprednisolone was associated with a more favorable 90‐day ordinal mRS distribution (adjusted common OR, 1.59; 95% CI, 1.11–2.28), higher odds of mRS 0–3 and 0–4, and lower mortality (aOR, 0.60; 95% CI, 0.37–0.96) and pneumonia (aOR, 0.61; 95% CI, 0.40–0.93), without an apparent increase in hemorrhage or gastrointestinal bleeding. No clear benefit was observed in patients with WBC < 10 × 10^9^/L.

**Conclusions:**

Higher admission WBC was associated with more favorable estimated treatment effects from adjunctive methylprednisolone after thrombectomy, warranting prospective validation.

**Trial Registration:** Chinese Clinical Trial Registry (ChiCTR.org.cn); ChiCTR2100051729; https://www.chictr.org.cn/

## Graphical abstract

Admission WBC identified who benefited from adjunctive methylprednisolone after thrombectomy. Improved 90‐day function and lower mortality/pneumonia occurred only with WBC ≥ 10 × 10^9^/L, supporting WBC‐guided corticosteroid targeting.

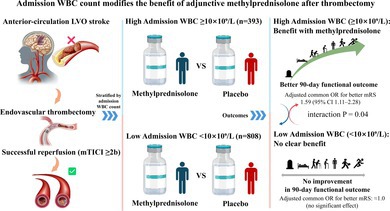

## 1 Introduction

Endovascular thrombectomy (EVT) is the standard of care for symptomatic anterior‐circulation large‐vessel occlusion (LVO) stroke [1, 2, 3, 4]. Despite high angiographic success (eTICI ≥ 2b in > 80% of routine cases), only approximately 30%–50% of patients achieve 90‐day functional independence, indicating a reperfusion–outcome mismatch in a substantial subset [5, 6, 7]. Inflammation‐mediated blood–brain barrier (BBB) injury has been implicated as a potential driver of this gap [8, 9].

Ischemia rapidly triggers neuroimmune crosstalk with systemic leukocyte activation and trafficking into the brain [10, 11]. While restrained responses may aid recovery, excessive activation disrupts the BBB, leading to edema, hemorrhagic complications, and is associated with poorer outcomes [9, 12]. These observations support testing time‐sensitive, postreperfusion pharmacologic modulation of inflammatory pathways.

Glucocorticoids (e.g., methylprednisolone) suppress pro‐inflammatory signaling, limit leukocyte adhesion/infiltration, and stabilize the endothelium partly by preserving junctional proteins and constraining matrix metalloproteinase activity [13, 14]. Experimental effects on edema vary by model and dose [15, 16], and clinical signals after EVT have been heterogeneous. In the MARVEL randomized trial, signals of benefit were greater for mortality and symptomatic hemorrhage outcomes (both statistically significantly lower in the methylprednisolone arm) than for distribution of disability levels (nonsignificantly favorable in methylprednisolone arm), suggesting that net effects may depend on the baseline inflammatory tone [17]. The admission white blood cell (WBC) count is an inexpensive, reproducible bedside index of systemic inflammation. Higher WBC has been linked to edema, hemorrhagic transformation, and unfavorable outcomes in EVT cohorts [12, 18].

Accordingly, for the present post hoc analysis, admission WBC was specified as the primary effect modifier, and we evaluated whether the estimated effect of adjunctive methylprednisolone varied by WBC stratum among patients with successful reperfusion (eTICI ≥ 2b). The primary outcome was the 90‐day ordinal modified Rankin Scale (mRS 0–6). We hypothesized that patients with elevated WBC (≥ 10 × 10^9^/L) would show more favorable treatment estimates than those with lower counts.

## 2 Methods

### 2.1 Study Design and Data Source

This post hoc secondary analysis used data from the MARVEL randomized, double‐blind, placebo‐controlled, multicenter trial [17], with details described in the published protocol [19] (ChiCTR2100051729). The statistical analysis plan for the present secondary analysis was prospectively finalized and time‐stamped before outcome modeling. In MARVEL, 1687 patients with anterior‐circulation large‐vessel occlusion (LVO) acute ischemic stroke undergoing endovascular thrombectomy (EVT) were randomized across 82 hospitals in China (2022–2023). After exclusion of 7 patients who withdrew consent immediately after randomization, 1680 patients were included in the parent trial primary analysis. Patients were randomized 1:1 to intravenous methylprednisolone sodium succinate (MPSS; 2 mg/kg/day for 3 days, initiated before EVT or within 2 h after EVT completion) or placebo. The trial's primary endpoint was the 90‐day distribution of the modified Rankin Scale (mRS 0–6).

### 2.2 Participants

Eligibility criteria have been reported previously. Briefly, adults (≥ 18 years) with intracranial internal carotid artery or M1/M2 middle cerebral artery occlusion treated with EVT within 24 h from last known well were eligible. Major exclusions included prestroke disability (mRS > 2), active infection requiring systemic therapy, and chronic corticosteroid or immunosuppressant use.

**FIGURE 1:**
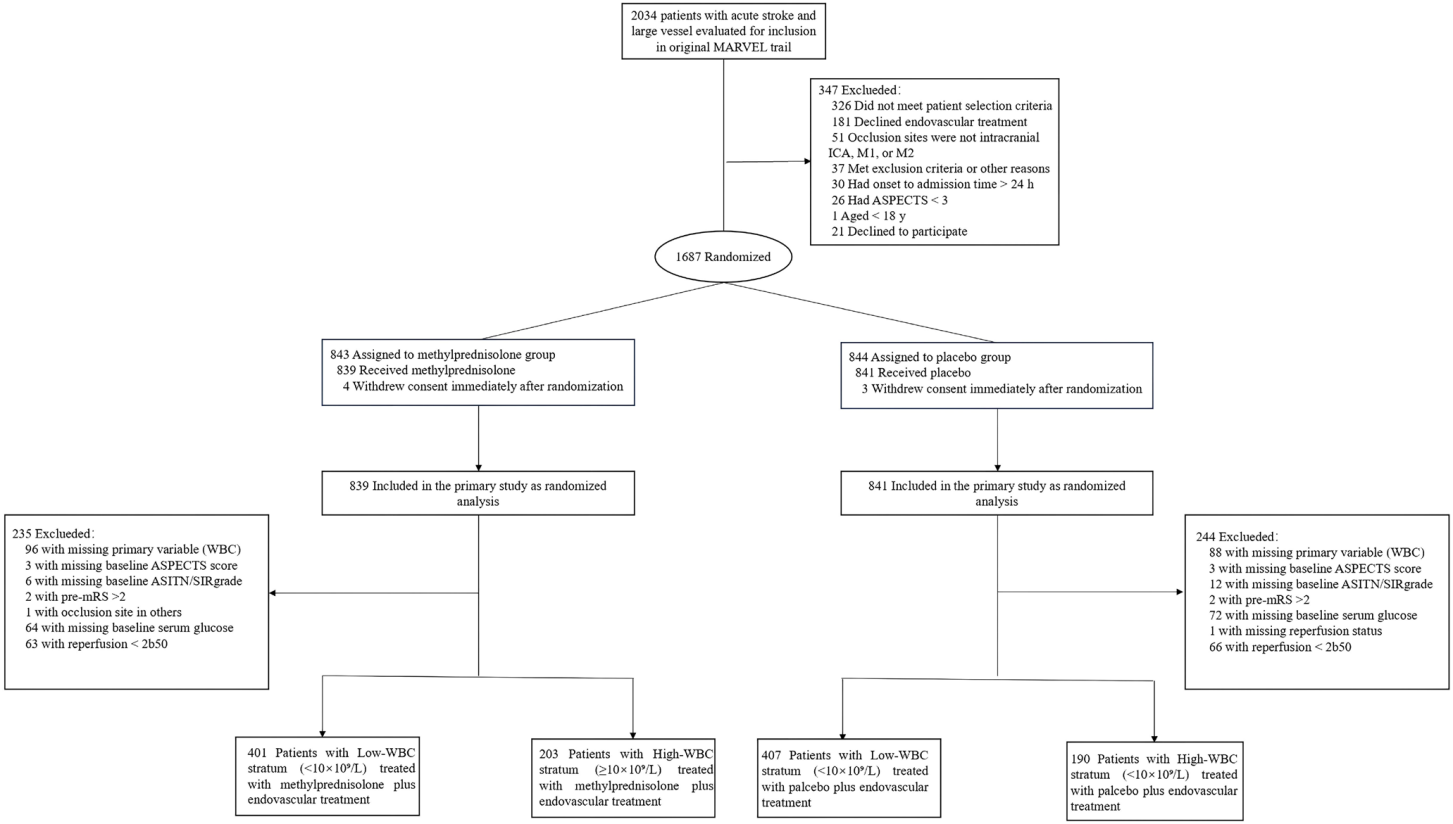
Study flow for the post hoc secondary analysis of the MARVEL Trial. In the MARVEL trial, 1687 patients were randomized (843 to methylprednisolone sodium succinate and 844 to placebo). After 7 patients withdrew consent immediately after randomization, 1680 patients were included in the parent trial primary analysis. Of these, 1496 had admission white blood cell (WBC) data. After excluding patients with missing baseline covariates, missing final reperfusion status, or unsuccessful reperfusion (final expanded Thrombolysis in Cerebral Infarction [eTICI] grade < 2b), 1201 patients were included in the baseline‐covariate complete‐case cohort and stratified by admission WBC (< 10 × 10^9^/L, *n* = 808; ≥ 10 × 10^9^/L, *n* = 393). AIS, acute ischemic stroke; ASITN/SIR, American Society of Interventional and Therapeutic Neuroradiology/Society of Interventional Radiology collateral grade; ASPECTS, Alberta Stroke Program Early CT Score; eTICI, expanded Thrombolysis in Cerebral Infarction; EVT, endovascular thrombectomy; ICA, internal carotid artery; MPSS, methylprednisolone sodium succinate; mRS, modified Rankin Scale; WBC, white blood cell count.

For this analysis, we restricted the cohort to patients with: (1) documented baseline WBC; and (2) successful reperfusion (expanded Thrombolysis in Cerebral Infarction, eTICI ≥ 2b); and (3) no missing baseline covariates. Study flow, exclusions, and cohort counts are shown in Figure 1.

The primary analysis used a baseline‐covariate complete‐case cohort of EVT‐treated patients with successful reperfusion and available baseline WBC. A multiple‐imputation (MI) sensitivity analysis imputed baseline covariates (*m* = 5); all outcomes were analyzed as observed. The MARVEL‐aligned covariate set specified in the analysis plan was applied in both analyses.

### 2.3 Baseline Variables and Measurements

Baseline variables included demographics (age, sex); vascular risk factors/comorbidities (hypertension, diabetes, atrial fibrillation, hyperlipidemia, smoking); vital signs (admission systolic/diastolic blood pressure); laboratory indices (admission serum glucose; white blood cell count [WBC]; lymphocyte count [LC]); stroke severity by the National Institutes of Health Stroke Scale (NIHSS) [20]. The stroke etiology followed the Trial of Org 10172 in Acute Stroke Treatment (TOAST) criteria [21]. Imaging scores/grades (Alberta Stroke Program Early CT Score, ASPECTS [22]; collateral grade per ASITN/SIR [23]); occlusion site (intracranial internal carotid artery, M1, M2); anesthesia type; workflow times (onset‐to‐puncture [OTP], puncture‐to‐reperfusion [PTR]); and intravenous thrombolysis as bridging therapy.

Admission WBC (×10^9^/L) was abstracted from routine clinical laboratories and analyzed as a continuous variable; for the primary effect‐modification analysis, WBC was dichotomized at < 10 vs. ≥ 10 × 10^9^/L, consistent with prior literature [24, 25].

### 2.4 Outcomes

The primary outcome was the 90‐day ordinal mRS (0–6). Secondary outcomes included functional thresholds (mRS 0–1, 0–2, 0–3, 0–4; multiplicity controlled with Holm–Bonferroni within this family), neurologic impairment (NIHSS at 5–7 days), and health status (EQ‐5D‐VAS at 90 days). Safety outcomes were all‐cause mortality at 90 days; symptomatic intracranial hemorrhage (sICH; Heidelberg Bleeding Classification [26]), any intracranial hemorrhage, pneumonia, and gastrointestinal bleeding within 7 days after EVT.

### 2.5 Statistical Analysis

Baseline characteristics within each WBC stratum (< 10 × 10^9^/L vs. ≥ 10 × 10^9^/L) are reported as median (IQR) or No. (%). Between‐group balance was quantified with standardized mean differences (SMD); **|SMD| < 0.10** denoted adequate balance. The primary analysis cohort was a baseline‐covariate complete‐case cohort, defined by available admission WBC and complete adjustment covariates specified in the analysis plan. Clinical outcomes were analyzed as observed and were not imputed. For each outcome, regression models included patients with available data for that specific outcome.

Effect modification by admission WBC (< 10 × 10^9^/L vs. ≥ 10 × 10^9^/L) was specified for the present post hoc analysis. The 90‐day ordinal mRS (0–6) was modeled with proportional‐odds logistic regression to estimate aCOR (> 1 favors MPSS). Binary outcomes used multivariable logistic regression to estimate aOR (> 1 favors MPSS for functional thresholds; < 1 for mortality/hemorrhagic endpoints). NIHSS at 5–7 days and EQ‐5D‐VAS at 90 days used linear regression to estimate adjusted mean differences (*β*). Effect modification was tested by including treatment, WBC stratum, and the treatment × WBC interaction (Wald tests, two‐sided *α* = 0.05). Within each WBC stratum, models adjusted for the MARVEL‐aligned covariate set: age, prestroke mRS, baseline NIHSS, baseline ASPECTS, intravenous thrombolysis, onset‐to‐randomization time, and occlusion site. Additional methodological details are provided in the Supporting Information Appendix in numerical order as Methods 1–6: Methods 1, statistical details and model specification; Methods 2, propensity‐score matching; Methods 3, analysis sets and covariates; Methods 4, multiple imputation; Methods 5, subgroup analyses; and Methods 6, the time‐stamped statistical analysis plan for the present post hoc secondary analysis. Supplementary visualizations are presented in the Supporting Information Appendix in numerical order as follows: covariate balance before and after matching (Figure S1), LASSO cross‐validation error curves and coefficient paths (Figure S2), missing‐data patterns (Figure S3), and multiple‐imputation diagnostics (Figure S4).

Sensitivity analyses. Multiple imputation (MI) was applied to baseline covariates using MICE (*m* = 5; predictive mean matching for continuous, logistic for binary, cumulative‐logit for ordered); all outcomes were analyzed as observed (no imputation), and estimates were combined with Rubin's rules. Missingness patterns and imputation diagnostics are shown in Figures S3 and S4. As a robustness check, propensity‐score matching (PSM) was conducted within WBC strata using 1:1 nearest‐neighbor matching on the logit of the propensity score with a caliper of 0.10, without replacement, using the MARVEL‐aligned covariate set to estimate the propensity score; postmatch balance was reassessed by SMD, and outcome models applied double adjustment for covariates from this set with residual imbalance (|SMD| > 0.10). Exploratory LASSO‐based models (overall interaction and within strata) were conducted as consistency checks; the LASSO‐based covariate‐selection framework is detailed in Supporting Information Methods 3, and the cross‐validation error curves and coefficient paths are shown in Figure S2.

Multiplicity for the family of secondary functional thresholds (mRS 0–1/0–2/0–3/0–4) was controlled with the Holm–Bonferroni procedure (family‐wise *α* = 0.05) within each analysis set (primary and MI‐sensitivity). No multiplicity adjustment was applied to the primary ordinal outcome or to safety endpoints; interaction *p*‐values are reported as nominal.

Adjusted risk differences (aRD; percentage points) for key binary endpoints were estimated, within strata, by marginal standardization of multivariable logistic models with robust (sandwich) standard errors; aRDs are reported in Tables 2 and S2 and were not used for primary inference.

To assess heterogeneity, we tested the treatment × WBC interaction and conducted nonconfirmatory subgroup analyses informed by LASSO‐based variable selection.

Analyses were performed in R 4.3.2 (ordinal, MatchIt, mice, sandwich; glmnet for exploratory work in the Supplement), with SPSS 26.0 and GraphPad Prism 8 supporting data management and graphics. This study was conducted and reported in accordance with the Consolidated Standards of Reporting Trials (CONSORT) guidelines [27].

## 3 Results

### 3.1 Study Population and Baseline Characteristics

Of 1687 randomized patients in MARVEL, 1680 were included in the parent trial primary analysis after 7 patients withdrew consent immediately after randomization. Among these 1680 patients, 1496 (89.0%) had available admission WBC data. After excluding patients with missing baseline covariates or without successful reperfusion, the baseline‐covariate complete‐case primary cohort for the present secondary analysis comprised 1201 patients. Among these, 808 (67.3%) had low WBC (< 10 × 10^9^/L) and 393 (32.7%) had high WBC (≥ 10 × 10^9^/L). Compared with the low‐WBC stratum, the high‐WBC stratum was younger and more often male, had more hypertension and hyperlipidemia but less atrial fibrillation, coronary heart disease, and valvular disease, and showed more intracranial ICA occlusions and worse collateral grades; stroke severity, early ischemic change, and workflow times were broadly similar. Within each stratum, treatment groups (Placebo vs. MPSS) were largely comparable (Table 1). A complementary visualization of patient flow by baseline WBC stratum, treatment assignment, and 90‐day mRS is shown in Figure S5.

**TABLE 1:** Baseline characteristics by treatment within WBC strata.

Variables	No./total (%)				No./total (%)				
	Low‐WBC stratum (< 10 × 10^9^/L, *n* = 808)				High WBC stratum (≥ 10 × 10^9^/L, *n* = 393)				SMD^2^
	Total	Placebo group (*n* = 407)	MPSS group (*n* = 401)	SMD^1^	Total	Placebo group (*n* = 190)	MPSS group (*n* = 203)	SMD^1^	
Age, years, median (IQR)	70.0 (59.0–77.0)	70.0 (60.0–78.0)	70.0 (59.0–76.0)	0.14	66.0 (57.0–73.0)	66.0 (56.2–74.0)	66.0 (58.0–73.0)	0.04	0.26
Sex, male, *n* (%)	443 (54.8)	227 (55.8)	216 (53.9)	0.04	253 (64.4)	122 (64.2)	131 (64.5)	0.01	0.20
Glucose, mmol/L, median (IQR)	7.0 (6.0–8.4)	6.8 (5.9–8.0)	7.3 (6.1–8.8)	0.15	7.3 (6.3–9.1)	7.4 (6.3–9.4)	7.1 (6.3–8.9)	0.07	0.10
WBC, ×10^9^/L, median (IQR)	7.4 (6.2–8.7)	7.4 (6.3–8.7)	7.5 (6.2–8.7)	0.04	12.1 (11.0–13.9)	12.0 (10.9–13.9)	12.5 (11.0–13.9)	0.02	2.60
Lymphocyte count, ×10^9^/L, median (IQR)	1.3 (0.9–1.8)	1.3 (0.9–1.8)	1.3 (0.8–1.8)	0.02	1.2 (0.8–1.7)	1.2 (0.9–1.8)	1.0 (0.7–1.7)	0.19	0.06
BP, mmHg, median (IQR)									
Systolic	144.0 (129.0–160.0)	145.0 (127.0–160.0)	144.0 (130.0–162.0)	0.04	145.0 (126.0–162.0)	146.0 (127.2–162.8)	143.0 (125.0–160.0)	0.05	0.02
Diastolic	83.0 (74.0–94.0)	83.0 (73.5–93.0)	82.0 (74.0–94.0)	0.00	85.0 (74.0–96.0)	87.0 (76.2–96.8)	83.0 (73.5–92.5)	0.16	0.05
Medical history, *n* (%)									
Hypertension	480 (59.4)	249 (61.2)	231 (57.6)	0.07	264 (67.2)	130 (68.4)	134 (66.0)	0.05	0.16
Hyperlipidemia	234 (29.0)	109 (26.8)	125 (31.2)	0.10	147 (37.4)	76 (40.0)	71 (35.0)	0.10	0.18
Diabetes	147 (18.2)	68 (16.7)	79 (19.7)	0.08	78 (19.8)	38 (20.0)	40 (19.7)	0.01	0.04
Smoking	218 (27.0)	111 (27.3)	107 (26.7)	0.01	117 (29.8)	55 (28.9)	62 (30.5)	0.04	0.06
Atrial fibrillation	357 (44.2)	187 (45.9)	170 (42.4)	0.07	134 (34.1)	58 (30.5)	76 (37.4)	0.15	0.21
Coronary heart disease	166 (20.5)	84 (20.6)	82 (20.4)	0.01	47 (12.0)	26 (13.7)	21 (10.3)	0.10	0.23
Valvular heart disease	124 (15.3)	65 (16.0)	59 (14.7)	0.04	36 (9.2)	20 (10.5)	16 (7.9)	0.09	0.19
Pre–mRS				0.16				0.17	0.06
0	786 (97.3)	401 (98.5)	385 (96.0)		380 (96.7)	184 (96.8)	196 (96.6)		
1	16 (2.0)	4 (1.0)	12 (3.0)		11 (2.6)	4 (2.1)	7 (3.4)		
2	6 (0.7)	2 (0.5)	4 (1.0)		2 (0.5)	2 (1.1)	0 (0.0)		
ASPECTS, median (IQR)	6.0 (4.0–7.0)	6.0 (4.0–8.0)	5.0 (4.0–7.0)	0.08	6.0 (4.0–7.0)	5.0 (4.0–7.0)	6.0 (4.0–7.0)	0.02	0.05
NIHSS score, median (IQR)	19.0 (16.0–21.0)	19.0 (16.0–21.0)	18.0 (16.0–21.0)	0.04	19.0 (17.0–21.0)	19.0 (17.0–21.0)	19.0 (17.0–21.0)	0.07	0.09
ASITN/SIR collateral grade, *n* (%)				0.10				0.29	0.12
0–1	295 (36.5)	144 (35.4)	151 (37.7)		164 (41.7)	82 (43.2)	82 (40.4)		
2	293 (36.3)	143 (35.1)	150 (37.4)		124 (31.6)	48 (25.3)	76 (37.4)		
3–4	220 (27.2)	120 (29.5)	100 (24.9)		105 (26.7)	60 (31.6)	45 (22.2)		
TOAST, *n* (%)				0.11				0.15	0.22
LAA	291 (36.0)	157 (38.6)	134 (33.4)		169 (43.0)	89 (46.8)	80 (39.4)		
CE	422 (52.2)	204 (50.1)	218 (54.4)		163 (41.5)	73 (38.4)	90 (44.3)		
Others or undetermined	95 (11.8)	46 (11.3)	49 (12.2)		61 (15.5)	28 (14.7)	33 (16.3)		
Occlusion site, *n* (%)				0.18				0.09	0.08
Internal carotid	265 (32.8)	131 (32.2)	134 (33.4)		143 (36.4)	73 (38.4)	70 (34.5)		
M1 segment	441 (54.6)	213 (52.3)	228 (56.9)		203 (51.7)	94 (49.5)	109 (53.7)		
M2 segment	102 (12.6)	63 (15.5)	39 (9.7)		47 (12.0)	23 (12.1)	24 (11.8)		
IVT, *n* (%)	302 (37.4)	161 (39.6)	141 (35.2)	0.09	134 (34.1)	71 (37.4)	63 (31.0)	0.13	0.07
General anesthesia, *n* (%)	563 (69.7)	285 (70.0)	278 (69.3)	0.02	275 (70.0)	135 (71.1)	140 (69.0)	0.05	0.01
Onset‐to‐randomization time, min, median (IQR)	353.5 (230.0–590.0)	357.0 (229.0–574.0)	348.0 (232.0–597.0)	0.01	387.0 (272.0–629.0)	387.0 (283.0–628.0)	381.0 (257.5–627.0)	0.04	0.04
Onset‐to‐puncture time, min, median (IQR)	345.0 (226.0–606.5)	351.0 (225.0–576.0)	340.0 (230.0–622.0)	0.01	384.0 (260.0–610.0)	392.0 (275.2–620.0)	380.0 (245.0–600.0)	0.02	0.03
Puncture‐to‐reperfusion time, min, median (IQR)	64.0 (39.0–107.0)	62.0 (40.0–109.5)	65.0 (39.0–105.0)	0.02	70.0 (40.0–108.0)	71.0 (41.2–107.2)	68.5 (40.0–112.0)	0.03	0.08

*Note:* Data presentation. Values are No. (%) unless otherwise indicated; continuous variables are reported as median (IQR) using an en‐dash. Balance metrics. SMD1 = standardized mean difference between Placebo and MPSS within each WBC stratum (primary balance metric; |SMD1| ≥ 0.10 denotes meaningful imbalance). SMD2 = standardized mean difference between WBC strata (descriptive only; not used to assess within‐stratum balance). Population. Table reflects the baseline‐covariate complete‐case primary cohort with eTICI ≥ 2b, observed admission WBC, and complete adjustment covariates.

Abbreviations: ASITN/SIR, American Society of Interventional and Therapeutic Neuroradiology/Society of Interventional Radiology collateral grade; ASPECTS, Alberta Stroke Program Early CT Score; eTICI, expanded Thrombolysis in Cerebral Infarction; EVT, endovascular thrombectomy; ICA, internal carotid artery; IVT, intravenous thrombolysis; LC, lymphocyte count; MCA, middle cerebral artery; MPSS, methylprednisolone sodium succinate; NIHSS, National Institutes of Health Stroke Scale; OTP, onset‐to‐puncture time; OTR, onset‐to‐randomization time; PTR, puncture‐to‐reperfusion time; SBP/DBP, systolic/diastolic blood pressure; WBC, white blood cell count.

### 3.2 Treatment Estimates Favored Adjunctive MPSS in Patients With WBC ≥ 10 × 10^9^/L

**FIGURE 2:**
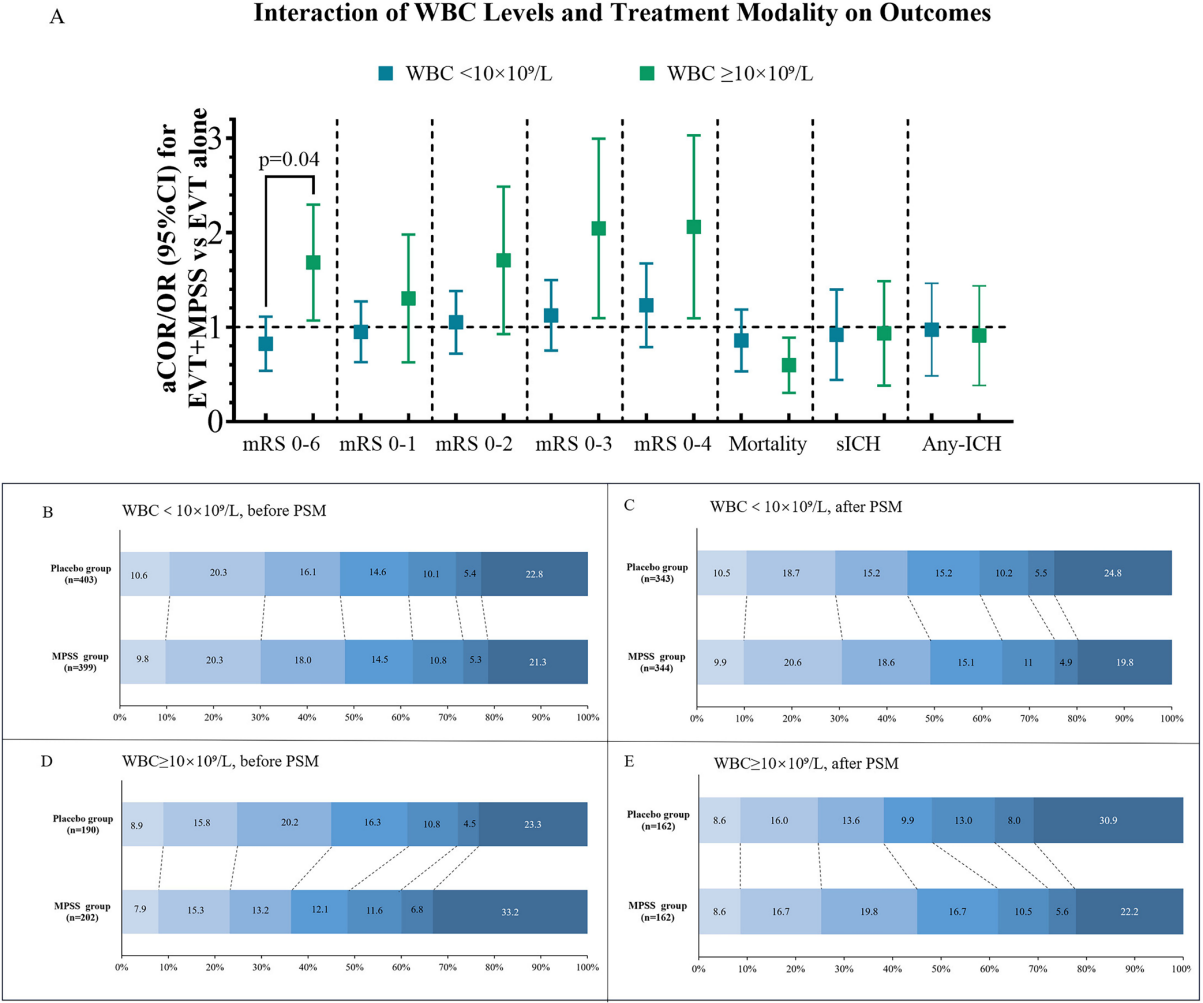
WBC‐stratified effects of adjunctive methylprednisolone after successful reperfusion. (A) Adjusted effect estimates comparing methylprednisolone sodium succinate (MPSS) versus placebo within strata of admission white blood cell count (WBC < 10 × 10^9^/L vs. ≥ 10 × 10^9^/L) across outcomes. Adjusted common odds ratios (aCORs) are shown for ordinal functional outcomes, and adjusted odds ratios (aORs) for binary outcomes. For functional outcomes, values > 1 indicate better outcomes with MPSS; for adverse outcomes (mortality, sICH, and any intracranial hemorrhage), values < 1 indicate lower risk with MPSS. The *p*‐value shown denotes the global treatment × WBC interaction (primary analysis); the multiple‐imputation sensitivity analysis yielded a similar interaction (*p* = 0.046). (B–E) Distribution of 90‐day modified Rankin Scale (mRS 0–6) by treatment before and after propensity score matching (PSM), shown separately for WBC < 10 × 10^9^/L (B, C) and WBC ≥ 10 × 10^9^/L (D, E). Bars represent row‐normalized percentages; sample sizes are indicated in each panel. Colors in panels (B–E) indicate mRS scores from 0 to 6, with darker shades representing higher disability; mRS 6 indicates death. Sample sizes differ slightly from Table 1 and Figure 1 because of missing 90‐day mRS assessments (3 Placebo and 2 MPSS in the low‐WBC stratum; 1 MPSS in the high‐WBC stratum). ACOR, adjusted common odds ratio; aOR, adjusted odds ratio; CI, confidence interval; ICH, intracranial hemorrhage; MPSS, methylprednisolone sodium succinate; mRS, modified Rankin Scale; PSM, propensity score matching; sICH, symptomatic intracranial hemorrhage; WBC, white blood cell count.

The treatment × WBC interaction reached nominal statistical significance (*p* = 0.04 in the complete‐case analysis; MI *p* = 0.046; Figure 2; Table S1). In the low‐WBC stratum (< 10 × 10^9/L), the 90‐day ordinal mRS did not differ between MPSS and Placebo (aCOR, 0.96; 95% CI, 0.75–1.23), and dichotomized functional outcomes, mortality, and sICH were likewise neutral (Table 2). In the high‐WBC stratum (≥ 10 × 10^9/L), MPSS was associated with a left‐shift of the mRS distribution (aCOR, 1.59; 95% CI, 1.11–2.28) and higher odds of mRS 0–3 (aOR, 1.86; 95% CI, 1.19–2.92) and 0–4 (aOR, 1.82; 95% CI, 1.15–2.88), which remained significant after Holm–Bonferroni adjustment within the mRS‐threshold family. Mortality was lower (aOR, 0.60; 95% CI, 0.37–0.96) and pneumonia within 7 days was reduced (aOR, 0.61; 95% CI, 0.40–0.93), with no increase in sICH (aOR, 0.81; 95% CI, 0.44–1.48). Rates of any intracranial hemorrhage and gastrointestinal bleeding showed no excess risk. On the absolute scale in the high‐WBC stratum, adjusted risk differences (percentage points) were: mRS 0–3, +14.7 (*p* = 0.01); mRS 0–4, +12.5 (*p* = 0.01); mortality, −9.7 (*p* = 0.04); pneumonia, −11.6 (*p* = 0.03); sICH, −1.7 (*p* = 0.58) (Table 2). Within‐treatment comparisons in Table S2 further contextualize these findings: in the Placebo cohort, high WBC was consistently associated with worse functional outcomes and higher mortality relative to low WBC, whereas in MPSS this adverse high‐vs‐low WBC gradient was largely attenuated, helping explain the observed treatment × WBC interaction. Figure 2 shows cumulative mRS distributions, illustrating a left‐shift with MPSS in the high‐WBC stratum. NIHSS at 5–7 days was not significantly different; EQ‐5D‐VAS was directionally consistent (Table 2). Additional details are provided in Table 2 and Table S2.

**TABLE 2:** Clinical outcomes by treatment, stratified by admission white blood cell (WBC) count.

	No./total (%)				No./total (%)			
	Low‐WBC stratum (< 10 × 10^9^/L, *n* = 808)				High‐WBC stratum (≥ 10 × 10^9^/L, *n* = 393)			
	Placebo group (*n* = 407)	MPSS group (*n* = 401)	Adjusted value (95% CI)	*p*	Placebo group (*n* = 190)	MPSS group (*n* = 203)	Adjusted value (95% CI)	*p*
Primary efficacy outcome *a*								
mRS score at 90 days, median (IQR)	3.0 (1.0 to 5.0)	3.0 (1.0 to 5.0)	aCOR: 0.96 (0.75 to 1.23)	0.75	4.0 (2.0 to 6.0)	3.0 (2.0 to 5.0)	aCOR: 1.59 (1.11 to 2.28)	0.01
Secondary efficacy outcomes *b*								
mRS 0–1 at 90 days	125/404 (30.9)	120/399 (30.1)	aOR: 0.91 (0.66 to 1.27)	0.58*	44/190 (23.2)	50/202 (24.8)	aOR: 1.12 (0.67 to 1.85)	0.67*
			aRD: −2.40 (−9.10 to 4.30)	0.48			aRD: 1.89 (−7.16 to 10.95)	0.68
mRS 0–2 at 90 days	190/404 (47.0)	192/399 (48.1)	aOR: 1.01 (0.74 to 1.37)	0.96*	69/190 (36.3)	91/202 (45.0)	aOR: 1.54 (0.98 to 2.41)	0.06*
			aRD: 0.09 (−7.56 to 7.75)	0.98			aRD: 10.62 (−0.30 to 21.55)	0.06
mRS 0–3 at 90 days	249/404 (61.6)	250/399 (62.7)	aOR: 1.01 (0.73 to 1.39)	0.97*	92/190 (48.4)	124/202 (61.4)	aOR: 1.86 (1.19 to 2.92)	0.01*
			aRD: 0.06 (−6.97 to 7.10)	0.99			aRD: 14.66 (3.99 to 25.34)	0.01
mRS 0–4 at 90 days	290/404 (71.8)	293/399 (73.4)	aOR: 1.09 (0.77 to 1.55)	0.63*	114/190 (60.0)	146/202 (72.3)	aOR: 1.82 (1.15 to 2.87)	0.01*
			aRD: 1.29 (−4.29 to 6.88)	0.65			aRD: 12.52 (2.88 to 22.16)	0.01
NIHSS score at 5–7 days, median (IQR)	9.5 (3.0 to 19.0)	10.0 (2.0 to 21.0)	*β*: 0.28 (−1.36 to 1.92)	0.74	13.0 (6.0 to 31.0)	11.0 (4.8 to 23.0)	*β*: −2.03 (−4.40 to 0.35)	0.10
EQ‐5D‐VAS score at 90 days, median (IQR)	60.0 (0.0 to 80.0)	55.0 (5.0 to 85.0)	*β*: −0.65 (−5.14 to 3.84)	0.78	30 (0 to 80)	55 (5 to 80)	*β*: 8.06 (1.38 to 14.75)	0.02
Safety outcomes								
Mortality	92/404 (22.8)	85/399 (21.3)	aOR: 0.91 (0.62 to 1.32)	0.61	63/190 (33.2)	47/202 (23.3)	aOR: 0.60 (0.37 to 0.96)	0.03
			aRD: −1.31 (−6.06 to 3.44)	0.59			aRD: −9.70 (−18.81 to −0.60)	0.04
Symptomatic intracranial hemorrhage	35/403 (8.7)	31/393 (7.9)	aOR: 0.89 (0.53 to 1.50)	0.67	28/189 (14.8)	25/202 (12.4)	aOR: 0.81 (0.44 to 1.48)	0.49
			aRD: −0.66 (−4.14 to 2.83)	0.71			aRD: −1.69 (−7.62 to 4.24)	0.58
Any radiologic intracranial hemorrhage	137/403 (34.0)	133/393 (33.8)	aOR: 0.99 (0.73 to 1.34)	0.94	81/189 (42.9)	101/202 (50.0)	aOR: 1.41 (0.93 to 2.10)	0.10
			aRD: −0.26 (−6.93 to 6.40)	0.94			aRD: 8.10 (−2.03 to 18.12)	0.12
Pneumonia	197/407 (48.4)	170/401 (42.4)	aOR: 0.81 (0.60 to 1.08)	0.15	116/190 (61.1)	101/203 (49.8)	aOR: 0.61 (0.40 to 0.93)	0.02
			aRD: −5.41 (−12.61 to 1.80)	0.15			aRD: −11.58 (−21.76 to −1.40)	0.03
Gastrointestinal bleeding within 7 days after EVT	21/407 (5.2)	12/401 (3.0)	aOR: 0.51 (0.24 to 1.08)	0.08	18/190 (9.5)	13/203 (6.4)	aOR: 0.64 (0.30 to 1.36)	0.25
			aRD: −1.67 (−3.64 to 0.30)	0.10			aRD: −3.04 (−8.36 to 2.28)	0.26

*Note:* Denominators and missing outcomes. Totals in column headers reflect the baseline‐covariate complete‐case primary cohort (*n* = 1201), defined by available admission WBC and complete adjustment covariates specified in the analysis plan. Clinical outcomes were analyzed as observed and were not imputed. Denominators in each row reflect patients with available data for the corresponding outcome and represent the exact sample size used in the corresponding regression model. The primary 90‐day ordinal mRS model included 1195 patients with available mRS data: 404 placebo and 399 MPSS in the low‐WBC stratum, and 190 placebo and 202 MPSS in the high‐WBC stratum. Primary endpoint. The 90‐day ordinal mRS (0–6) was analyzed with a proportional‐odds model; effects are reported as adjusted common odds ratios (aCORs), with values > 1.00 favoring MPSS. Binary endpoints. Multivariable logistic regression was used to estimate adjusted odds ratios (aORs). Adjusted risk differences (aRDs; percentage points) were derived via marginal standardization with robust standard errors. Continuous endpoints. Linear regression was used to estimate adjusted mean differences (*β*). Covariates. Models were adjusted for age, prestroke mRS score, baseline NIHSS score, baseline ASPECTS, use of intravenous thrombolysis, onset‐to‐randomization time, and occlusion site, consistent with the primary MARVEL analysis. Multiplicity. Holm–Bonferroni adjustment was applied separately within each WBC stratum to the family of four dichotomized functional thresholds: mRS 0–1, 0–2, 0–3, and 0–4. The two WBC strata were not pooled for this multiplicity adjustment. Reported table *p‐*values are nominal. Holm‐adjusted *p‐*values were as follows: low‐WBC stratum, all four thresholds had adjusted *p* > 0.99; high‐WBC stratum, mRS 0–1, *p* = 0.67; mRS 0–2, *p* = 0.12; mRS 0–3, *p* = 0.04; and mRS 0–4, *p* = 0.04. The primary ordinal endpoint and safety endpoints were not multiplicity‐adjusted. Directionality. For functional outcomes, aOR > 1.00 or aRD > 0 favors MPSS. For safety outcomes, aOR < 1.00 or aRD < 0 favors MPSS. For NIHSS, lower values are better; for EQ‐5D‐VAS, higher values are better. Reporting. Effect sizes are shown with 95% CIs; two‐sided *p‐*values are reported.

Abbreviations: aCOR, adjusted common odds ratio; aOR, adjusted odds ratio; aRD, adjusted risk difference; CI, confidence interval; IQR, interquartile range; MPSS, methylprednisolone sodium succinate; mRS, modified Rankin Scale; NIHSS, National Institutes of Health Stroke Scale; sICH, symptomatic intracranial hemorrhage; VAS, visual analog scale; WBC, white blood cell; *β*, adjusted mean difference.

* symbol indicates the Holm–Bonferroni adjusted *p* values for the dichotomized functional thresholds (mRS 0–1, 0–2, 0–3, 0–4) within each WBC stratum.

### 3.3 Robustness and Consistency

In propensity‐score–matched cohorts, most covariates achieved adequate postmatch balance, and residual imbalances were addressed by double adjustment in the outcome models (Table S3). Covariate balance before and after matching is visually summarized in Figure S1. Findings remained neutral across endpoints in the low‐WBC group, whereas in the high‐WBC group MPSS was associated with a more favorable ordinal mRS (aCOR, 1.50; 95% CI, 1.00–2.23), increased the odds of mRS 0–3 and 0–4, reduced mortality (aOR, 0.57; 95% CI, 0.33–0.99) and pneumonia within 7 days (aOR, 0.54; 95% CI, 0.34–0.86), and did not increase sICH (aOR, 0.65; 95% CI, 0.31–1.33) (Table S4). Multiple‐imputation analyses were concordant—neutral in low WBC and favorable in high WBC (high‐WBC ordinal mRS aCOR, 1.53; 95% CI, 1.09–2.15; Table S6). Exploratory LASSO‐based models showed similar directions of effect (Table S5).

### 3.4 Subgroup Analyses in Patients With WBC ≥ 10 × 10^9^/L

**FIGURE 3:**
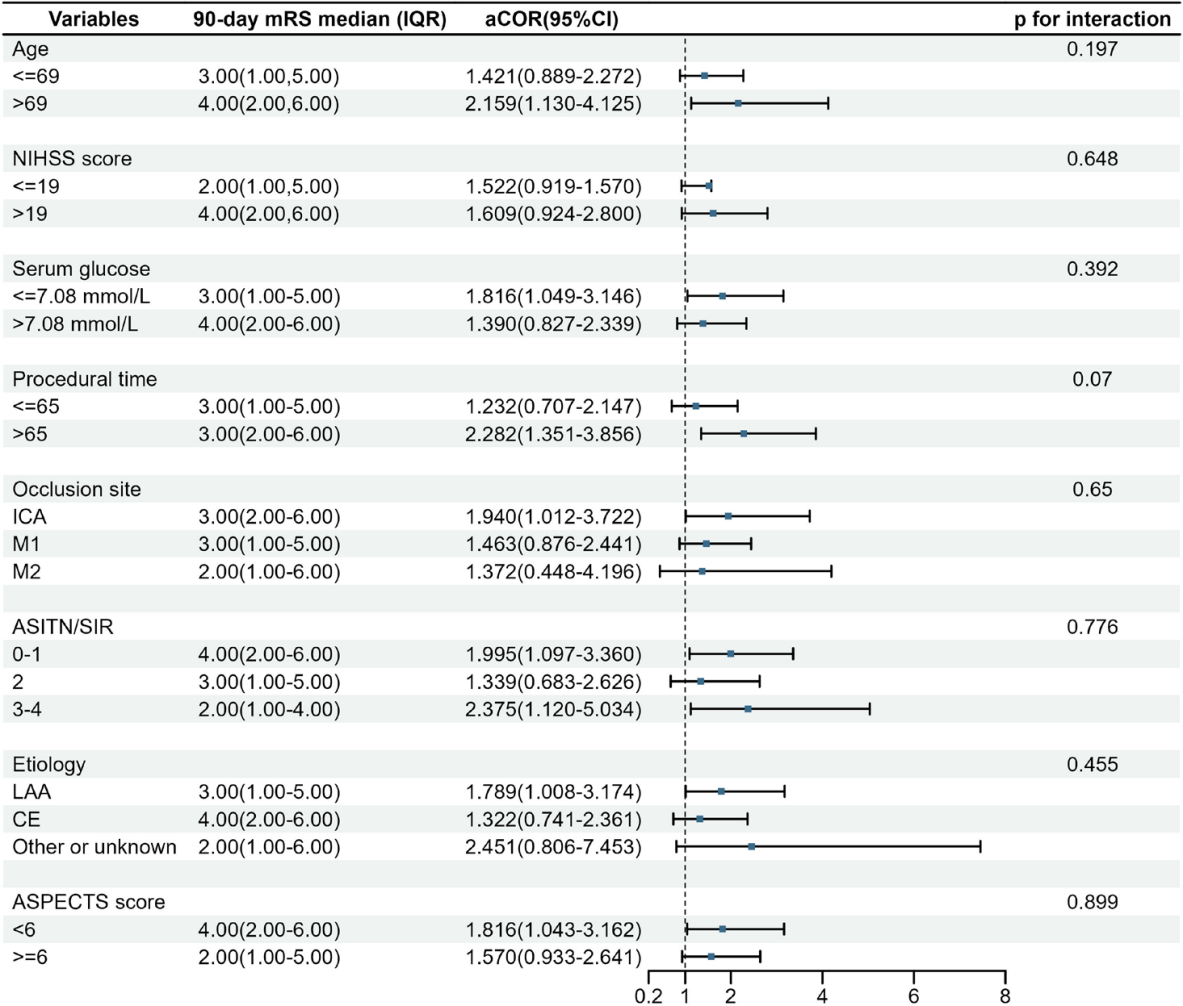
Subgroup analyses within the high‐WBC stratum. Forest plot of adjusted common odds ratios (aCORs) for the association of methylprednisolone sodium succinate (MPSS) vs. placebo with 90‐day ordinal modified Rankin Scale (mRS) among patients with admission WBC ≥ 10 × 10^9^/L. Points indicate aCORs and horizontal lines indicate 95% CIs from covariate‐adjusted proportional‐odds models using the same MARVEL‐aligned covariate set as in the primary analysis. For the ordinal mRS shift, aCORs > 1 indicate better outcomes with MPSS. The vertical dashed line indicates no effect (aCOR = 1). *p*‐values shown are for treatment‐by‐subgroup interaction and are exploratory (no multiplicity adjustment). ACOR, adjusted common odds ratio; ASITN/SIR, American Society of Interventional and Therapeutic Neuroradiology/Society of Interventional Radiology collateral grade; ASPECTS, Alberta Stroke Program Early CT Score; CI, confidence interval; ICA, internal carotid artery; M1/M2, first/s segment of the middle cerebral artery; MPSS, methylprednisolone sodium succinate; mRS, modified Rankin Scale; NIHSS, National Institutes of Health Stroke Scale; TOAST, Trial of Org 10172 in Acute Stroke Treatment.

Within the high‐WBC stratum, the association of MPSS with better outcomes was broadly consistent across subgroups (Figure 3; all interaction *p* > 0.05).

## 4 Discussion

This post hoc secondary analysis of the MARVEL randomized trial, conducted with a prospectively finalized and time‐stamped statistical analysis plan, is, to our knowledge, among the first to evaluate adjunctive MPSS in AIS patients stratified by an inflammatory biomarker. Admission WBC was associated with a differential pattern of estimated treatment effects: among patients with WBC ≥ 10 × 10^9^/L, MPSS was associated with more favorable functional outcomes and lower mortality without an apparent increase in sICH, whereas no clear benefit was observed in patients with lower WBC. Beyond disability and mortality, the high‐WBC stratum also showed lower early pneumonia and no excess intracranial hemorrhage or gastrointestinal bleeding. Accordingly, a WBC threshold of ≥ 10 × 10^9^/L provides a pragmatic candidate marker for treatment enrichment, identifying patients more likely to benefit from adjunctive MPSS while helping avoid indiscriminate exposure in those with low inflammatory burden. Prospective trials are warranted to confirm and refine this stratification strategy.

Evidence on corticosteroids for AIS has been largely neutral amid heterogeneity in treatment context, timing, and dosing regimens [25, 28]. In EVT‐treated populations, MARVEL showed no overall difference in the primary 90‐day ordinal mRS [17], and a contemporary systematic review suggested a possible short‐term mortality reduction with uncertain functional effects [29]. Importantly, prior studies did not incorporate stratification by inflammatory biomarkers or test a treatment × WBC interaction, leaving open whether inflammation‐enriched subgroups could benefit—an uncertainty addressed by the present WBC‐based analysis.

The observed treatment × WBC interaction suggests that the estimated effect of MPSS may vary according to baseline inflammatory burden. In patients with low WBC (< 10 × 10^9^/L), systemic inflammation is limited and successful EVT addresses the primary occlusive lesion, leaving little scope for incremental anti‐inflammatory benefit. Conversely, in patients with high WBC (≥ 10 × 10^9^/L)—indicative of a more pro‐inflammatory milieu and greater BBB vulnerability—MPSS may yield greater net benefit [30, 31]. This directional pattern was consistent across ordinal mRS levels and mortality, without a signal of increased sICH. Biologically, ischemia–reperfusion provokes sterile inflammation and BBB injury [30, 32], whereas glucocorticoids mitigate secondary injury by suppressing pro‐inflammatory signaling, inhibiting MMP activity, and stabilizing endothelial tight junctions (e.g., occludin/claudin‐5/VE‐cadherin) [33, 34, 35, 36]. Concordant preclinical studies in reperfusion‐relevant models further show reductions in infarct size, BBB permeability, and brain water content with MPSS, along with preservation of BBB integrity [37, 38, 39].

Within‐treatment comparisons also support an inflammation‐mediated pathway: among patients receiving Placebo, higher WBC was associated with worse outcomes than lower WBC, whereas among those receiving MPSS, higher WBC was not associated with worse outcomes. These findings suggest that, at a stratum level, the ≥ 10 × 10^9^/L state likely coincides with greater leukocyte–endothelium activation, adhesion/trafficking and chemotactic signaling, oxidative stress, and MMP‐linked BBB fragility—nodes aligned with glucocorticoid‐modifiable targets. Therefore, anti‐inflammatory and barrier‐stabilizing effects can translate into greater net clinical benefit. In contrast, when WBC < 10 × 10^9^/L, the post‐EVT inflammatory substrate for incremental modulation is limited. Taken together, these observations support a pragmatic, WBC‐guided stratification pathway that was consistent across analytic approaches. Future inflammation‐stratified trials, and the integration of other accessible biomarkers such as NLR or CRP [37, 40], may further refine selection without increasing workflow complexity.

In patients with admission WBC ≥ 10 × 10^9^/L, adjunctive methylprednisolone reduced mortality, lowered early pneumonia, and did not increase gastrointestinal bleeding; in WBC < 10 × 10^9^/L, functional/mortality benefits were not observed, yet pneumonia also declined without excess GI bleeding (Table 2; Table S2). For hemorrhagic endpoints, there was no overall rise in sICH; within the high‐WBC stratum, a nonsignificant decrease in sICH alongside a nonsignificant uptick in any ICH is compatible with a phenotypic shift: postreperfusion BBB stabilization may curtail space‐occupying hematomas that drive symptoms while leaving small petechial changes largely unaffected and potentially more frequently detected under intensive imaging—consistent with recent data on the prognostic divergence of HI versus PH categories [41, 42, 43]. This two‐stratum pattern aligns with a systemic, multi‐target anti‐inflammatory and vascular‐stabilizing program: glucocorticoids attenuate leukocyte–endothelium adhesion/rolling and adhesion‐molecule axes (selectins/integrins) [34, 44], suppress NF‐κB–driven cytokines (e.g., IL‐6, TNF‐α) [34, 45], and down‐regulate MMP‐9 while preserving tight‐junction proteins (occludin, claudin‐5, VE‐cadherin) to maintain barrier integrity [31, 35, 46]; in parallel, glycocalyx preservation and reduced systemic endothelial leak provide a coherent link to fewer pneumonias across strata [47, 48, 49], complemented by mitigation of brain–lung and gut–lung inflammatory cross‐talk [34, 50, 51]. Moreover, a short peri‐procedural course (2 mg/kg for 3 days) entails a shorter immunosuppressive window and lower infectious risk than prolonged/high‐dose regimens, in keeping with the pneumonia signal [52, 53]. Overall, the safety profile appears reassuring and mechanistically coherent; confirmation in prospective, biomarker‐stratified studies with standardized infection and bleeding surveillance and protocol‐defined hierarchical testing remains warranted.

Among patients with WBC ≥ 10 × 10^9^/L, MPSS showed broadly consistent associations across subgroups defined by age, ASPECTS, occlusion site, glucose, stroke etiology, and collateral status (ASITN/SIR 0–1 and 3–4). These findings suggest that elevated WBC may serve as a simple and practical candidate enrichment marker across diverse clinical profiles. Looking ahead, future work should clarify practical thresholds and temporal dynamics for inflammatory markers, refine joint stratification with key clinical/imaging features, and optimize treatment timing and dosing to delineate candidates most likely to benefit, while ongoing randomized trials in large‐core/low‐ASPECTS populations (NCT06360458; MATCH, NCT06870448) may help assess generalizability.

## 5 Limitations

This was a post hoc secondary analysis conducted with a prospectively finalized and time‐stamped statistical analysis plan. Restriction to successfully reperfused patients (eTICI ≥ 2b) strengthened internal validity for the adjunctive EVT context but may limit generalizability. Conducting the primary analysis in a baseline‐covariate complete‐case cohort (*n* = 1201) may introduce selection bias; however, findings were robust in multiple‐imputation sensitivity analyses. Admission WBC was obtained before randomization and the protocol excluded active infection, yet inter‐center measurement differences and potential effects of physiologic stress or occult infection could still influence WBC, leaving room for measurement error and residual confounding. Finally, all trial sites were in China, which may affect external validity. Despite these caveats, triangulation across covariate‐adjusted models, propensity‐score matching, and multiple imputation yielded consistent direction and magnitude of effects, supporting the consistency of the observed treatment‐estimate pattern, while prospective validation is required before admission WBC can be considered a predictive treatment marker.

## 6 Conclusions

In this post hoc secondary analysis of MARVEL restricted to successfully reperfused EVT patients, higher admission WBC identified a subgroup with more favorable estimated treatment effects from adjunctive methylprednisolone, whereas estimates were neutral in patients with lower WBC. No signal of increased sICH was observed. These findings support prospective evaluation of WBC‐guided enrichment strategies in future trials.

## Author Contributions

Concept and design were contributed by Wenjie Zi, Kunxin Lin. Acquisition, analysis, or interpretation of data by Chengsong Yue, Jie Yang, Dahong Yang, Guanting Heng, Linyu Li, Shihai Yang, Xuanyu Chen, Yi Lin, Wenjie Zi. Drafting of the manuscript by Chengsong Yue, Jie Yang, Dahong Yang, Guanting Heng, Kunxin Lin. Critical revision of the manuscript for important intellectual content by all authors. Statistical analysis by Chengsong Yue, Jie Yang, Wenjie Zi. Administrative, technical, or material support by Dahong Yang, Guanting Heng, Linyu Li, Shihai Yang, Xuanyu Chen, Yi Lin. Supervision by Wenjie Zi, Kunxin Lin.

## Funding

This secondary analysis was supported by the National Natural Science Foundation of China (NSFC) [No. 82425021].

## Disclosure

Dr. Zi had full access to all the data in the study and takes responsibility for the integrity of the data and the accuracy of the data analysis.

## Ethics Statement

The MARVEL trial received ethics approval from the institutional review boards of all participating centers (approval No. 2021‐yandi131‐01); written informed consent was obtained from all participants or their legally authorized representatives.

## Consent

This post hoc secondary analysis used deidentified data collected under the original protocol and consent.

## Conflicts of Interest

The authors declare no conflicts of interest.

## Supporting information


**Data S1:** Supporting Information.

## Data Availability

The data that support the findings of this study are available on request from the corresponding author. The data are not publicly available due to privacy or ethical restrictions.

## References

[1] W. J. Powers , A. A. Rabinstein , T. Ackerson , et al., “Guidelines for the Early Management of Patients With Acute Ischemic Stroke: 2019 Update to the 2018 Guidelines for the Early Management of Acute Ischemic Stroke: A Guideline for Healthcare Professionals From the American Heart Association/American Stroke Association,” Stroke 50, no. 12 (2019): e344–e418. 31662037 10.1161/STR.0000000000000211

[2] G. Turc , P. Bhogal , U. Fischer , et al., “European Stroke Organisation (ESO) ‐ European Society for Minimally Invasive Neurological Therapy (ESMINT) Guidelines on Mechanical Thrombectomy in Acute Ischemic Stroke,” Journal of NeuroInterventional Surgery 15, no. 8 (2023): e8. 30808653 10.1136/neurintsurg-2018-014569

[3] T. N. Nguyen , A. C. Castonguay , J. E. Siegler , et al., “Mechanical Thrombectomy in the Late Presentation of Anterior Circulation Large Vessel Occlusion Stroke: A Guideline From the Society of Vascular and Interventional Neurology Guidelines and Practice Standards Committee,” Stroke: Vascular and Interventional Neurology 3, no. 1 (2023): e000512. 39380893 10.1161/SVIN.122.000512 PMC11460660

[4] A. Sahoo , M. Abdalkader , H. Yamagami , et al., “Endovascular Therapy for Acute Stroke: New Evidence and Indications,” Journal of Neuroendovascular Therapy 17, no. 11 (2023): 232–242. 38025253 10.5797/jnet.ra.2023-0047 PMC10657733

[5] L. Winkelmeier , T. D. Faizy , G. Broocks , et al., “Association Between Recanalization Attempts and Functional Outcome After Thrombectomy for Large Ischemic Stroke,” Stroke 54, no. 9 (2023): 2304–2312. 37492970 10.1161/STROKEAHA.123.042794 PMC10464881

[6] X. Huo , D. Sun , Raynald , et al., “Endovascular Treatment in Acute Ischemic Stroke With Large Vessel Occlusion According to Different Stroke Subtypes: Data From ANGEL‐ACT Registry,” Neurology and Therapy 11, no. 1 (2022): 151–165. 34800279 10.1007/s40120-021-00301-z PMC8857367

[7] F. Seker , M. M. Qureshi , M. A. Möhlenbruch , et al., “Reperfusion Without Functional Independence in Late Presentation of Stroke With Large Vessel Occlusion,” Stroke 53, no. 12 (2022): 3594–3604. 36252092 10.1161/STROKEAHA.122.039476

[8] R. G. Nogueira , A. P. Jadhav , D. C. Haussen , et al., “Thrombectomy 6 to 24 Hours After Stroke With a Mismatch Between Deficit and Infarct,” New England Journal of Medicine 378, no. 1 (2018): 11–21. 29129157 10.1056/NEJMoa1706442

[9] L. Mechtouff , N. Debs , C. Frindel , et al., “Association of Blood Biomarkers of Inflammation With Penumbra Consumption After Mechanical Thrombectomy in Patients With Acute Ischemic Stroke,” Neurology 99, no. 18 (2022): e2063–e2071. 36316128 10.1212/WNL.0000000000201038

[10] C. Iadecola , M. S. Buckwalter , and J. Anrather , “Immune Responses to Stroke: Mechanisms, Modulation, and Therapeutic Potential,” Journal of Clinical Investigation 130, no. 6 (2020): 2777–2788. 32391806 10.1172/JCI135530 PMC7260029

[11] M. Duan , Y. Xu , Y. Li , H. Feng , and Y. Chen , “Targeting Brain‐Peripheral Immune Responses for Secondary Brain Injury After Ischemic and Hemorrhagic Stroke,” Journal of Neuroinflammation 21, no. 1 (2024): 102. 38637850 10.1186/s12974-024-03101-y PMC11025216

[12] D. Ferro , M. Matias , J. Neto , et al., “Neutrophil‐To‐Lymphocyte Ratio Predicts Cerebral Edema and Clinical Worsening Early After Reperfusion Therapy in Stroke,” Stroke 52, no. 3 (2021): 859–867. 33517702 10.1161/STROKEAHA.120.032130

[13] A. Simats and A. Liesz , “Systemic Inflammation After Stroke: Implications for Post‐Stroke Comorbidities,” EMBO Molecular Medicine 14, no. 9 (2022): e16269. 35971650 10.15252/emmm.202216269 PMC9449596

[14] F. Shimizu and M. Nakamori , “Blood‐Brain Barrier Disruption in Neuroimmunological Disease,” International Journal of Molecular Sciences 25, no. 19 (2024): 10625. 39408955 10.3390/ijms251910625 PMC11476930

[15] G. M. de Courten‐Myers , M. Kleinholz , K. R. Wagner , G. Xi , and R. E. Myers , “Efficacious Experimental Stroke Treatment With High‐Dose Methylprednisolone,” Stroke 25, no. 2 (1994): 487–492, discussion 493. 8303761 10.1161/01.str.25.2.487

[16] L. M. Barbosa‐Coutinho , A. Hartmann , K. A. Hossmann , and T. Rommel , “Effect of Dexamethasone on Serum Protein Extravasation in Experimental Brain Infarcts of Monkey: An Immunohistochemical Study,” Acta Neuropathologica 65, no. 3‐4 (1985): 255–260. 3883688 10.1007/BF00687005

[17] Investigators MTAftM , Q. Yang , C. Guo , et al., “Methylprednisolone as Adjunct to Endovascular Thrombectomy for Large‐Vessel Occlusion Stroke: The MARVEL Randomized Clinical Trial,” Journal of the American Medical Association 331, no. 10 (2024): 840–849. 38329440 10.1001/jama.2024.0626 PMC10853866

[18] A. Semerano , C. Laredo , Y. Zhao , et al., “Leukocytes, Collateral Circulation, and Reperfusion in Ischemic Stroke Patients Treated With Mechanical Thrombectomy,” Stroke 50, no. 12 (2019): 3456–3464. 31619153 10.1161/STROKEAHA.119.026743

[19] Q. Yang , C. Guo , C. Yue , et al., “MARVEL: A Randomized Double‐Blind, Placebo‐Controlled Trial in Patients Undergoing Endovascular Therapy: Study Rationale and Design,” Stroke: Vascular and Interventional Neurology 4, no. 2 (2024): e001090. 41583597 10.1161/SVIN.123.001090 PMC12778488

[20] T. Brott , H. P. Adams, Jr. , C. P. Olinger , et al., “Measurements of Acute Cerebral Infarction: A Clinical Examination Scale,” Stroke 20, no. 7 (1989): 864–870. 2749846 10.1161/01.str.20.7.864

[21] H. P. Adams, Jr. , B. H. Bendixen , L. J. Kappelle , et al., “Classification of Subtype of Acute Ischemic Stroke. Definitions for Use in a Multicenter Clinical Trial. TOAST. Trial of Org 10172 in Acute Stroke Treatment,” Stroke 24, no. 1 (1993): 35–41. 7678184 10.1161/01.str.24.1.35

[22] P. A. Barber , A. M. Demchuk , J. Zhang , and A. M. Buchan , “Validity and Reliability of a Quantitative Computed Tomography Score in Predicting Outcome of Hyperacute Stroke Before Thrombolytic Therapy. ASPECTS Study Group. Alberta Stroke Programme Early CT Score,” Lancet 355, no. 9216 (2000): 1670–1674. 10905241 10.1016/s0140-6736(00)02237-6

[23] R. T. Higashida , A. J. Furlan , H. Roberts , et al., “Trial Design and Reporting Standards for Intra‐Arterial Cerebral Thrombolysis for Acute Ischemic Stroke,” Stroke 34, no. 8 (2003): e109–e137. 12869717 10.1161/01.STR.0000082721.62796.09

[24] J. C. Furlan , M. D. Vergouwen , J. Fang , and F. L. Silver , “White Blood Cell Count Is an Independent Predictor of Outcomes After Acute Ischaemic Stroke,” European Journal of Neurology 21, no. 2 (2014): 215–222. 23848934 10.1111/ene.12233

[25] S. You , Z. Ou , W. Zhang , et al., “Combined Utility of White Blood Cell Count and Blood Glucose for Predicting In‐Hospital Outcomes in Acute Ischemic Stroke,” Journal of Neuroinflammation 16, no. 1 (2019): 37. 30764852 10.1186/s12974-019-1422-7 PMC6375165

[26] R. von Kummer , J. P. Broderick , B. C. Campbell , et al., “The Heidelberg Bleeding Classification: Classification of Bleeding Events After Ischemic Stroke and Reperfusion Therapy,” Stroke 46, no. 10 (2015): 2981–2986. 26330447 10.1161/STROKEAHA.115.010049

[27] K. F. Schulz , D. G. Altman , D. Moher , and Group C , “CONSORT 2010 Statement: Updated Guidelines for Reporting Parallel Group Randomised Trials,” Trials 11 (2010): 32. 21350618 10.4103/0976-500X.72352 PMC3043330

[28] P. A. Sandercock and T. Soane , “Corticosteroids for Acute Ischaemic Stroke,” Cochrane Database of Systematic Reviews 9 (2011): CD000064. 10.1002/14651858.CD000064.pub2 PMC7032681 21901674

[29] Y. Wang , L. Huang , J. Li , et al., “Efficacy and Safety of Corticosteroids for Stroke and Traumatic Brain Injury: A Systematic Review and Meta‐Analysis,” Systematic Reviews 14, no. 1 (2025): 54. 40038828 10.1186/s13643-025-02803-5 PMC11877790

[30] E. Candelario‐Jalil , R. M. Dijkhuizen , and T. Magnus , “Neuroinflammation, Stroke, Blood‐Brain Barrier Dysfunction, and Imaging Modalities,” Stroke 53, no. 5 (2022): 1473–1486. 35387495 10.1161/STROKEAHA.122.036946 PMC9038693

[31] E. Salvador , S. Shityakov , and C. Förster , “Glucocorticoids and Endothelial Cell Barrier Function,” Cell and Tissue Research 355, no. 3 (2014): 597–605. 24352805 10.1007/s00441-013-1762-z PMC3972429

[32] W. Abdullahi , D. Tripathi , and P. T. Ronaldson , “Blood‐Brain Barrier Dysfunction in Ischemic Stroke: Targeting Tight Junctions and Transporters for Vascular Protection,” American Journal of Physiology. Cell Physiology 315, no. 3 (2018): C343–C356. 29949404 10.1152/ajpcell.00095.2018 PMC6171039

[33] Q. Guo , Y. Jin , X. Chen , et al., “NF‐κB in Biology and Targeted Therapy: New Insights and Translational Implications,” Signal Transduction and Targeted Therapy 9, no. 1 (2024): 53. 38433280 10.1038/s41392-024-01757-9 PMC10910037

[34] K. A. Zielińska , L. Van Moortel , G. Opdenakker , K. De Bosscher , and P. E. Van den Steen , “Endothelial Response to Glucocorticoids in Inflammatory Diseases,” Frontiers in Immunology 7 (2016): 592. 28018358 10.3389/fimmu.2016.00592 PMC5155119

[35] K. A. Harkness , P. Adamson , J. D. Sussman , G. A. Davies‐Jones , J. Greenwood , and M. N. Woodroofe , “Dexamethasone Regulation of Matrix Metalloproteinase Expression in CNS Vascular Endothelium,” Brain 123, no. 4 (2000): 698–709. 10734001 10.1093/brain/123.4.698

[36] Y. Hashimoto , C. Greene , A. Munnich , and M. Campbell , “The CLDN5 Gene at the Blood‐Brain Barrier in Health and Disease,” Fluids and Barriers of the CNS 20, no. 1 (2023): 22. 36978081 10.1186/s12987-023-00424-5 PMC10044825

[37] A. Zietz , S. Gorey , P. J. Kelly , M. Katan , and J. J. McCabe , “Targeting Inflammation to Reduce Recurrent Stroke,” International Journal of Stroke 19, no. 4 (2024): 379–387. 37800305 10.1177/17474930231207777 PMC10964390

[38] P. Kozler , D. Marešová , and J. Pokorný , “Effect of Methylprednisolone on Experimental Brain Edema in Rats ‐ Own Experience Reviewed,” Physiological Research 70, no. S3 (2021): S289–S300. 35099248 10.33549/physiolres.934818 PMC8884394

[39] S. Cheng , W. Gao , X. Xu , et al., “Methylprednisolone Sodium Succinate Reduces BBB Disruption and Inflammation in a Model Mouse of Intracranial Haemorrhage,” Brain Research Bulletin 127 (2016): 226–233. 27746369 10.1016/j.brainresbull.2016.10.007

[40] D. Sharma , K. J. Spring , and S. Bhaskar , “Role of Neutrophil‐Lymphocyte Ratio in the Prognosis of Acute Ischaemic Stroke After Reperfusion Therapy: A Systematic Review and Meta‐Analysis,” Journal of Central Nervous System Disease 14 (2022): 11795735221092518. 35492740 10.1177/11795735221092518 PMC9052237

[41] C. H. Chen , A. Shoamanesh , P. Colorado , et al., “Hemorrhagic Transformation in Noncardioembolic Acute Ischemic Stroke: MRI Analysis From PACIFIC‐STROKE,” Stroke 55, no. 6 (2024): 1477–1488. 38690666 10.1161/STROKEAHA.123.045204

[42] M. K. Luff , N. Khezri , S. Miralbes , et al., “Hemorrhagic Transformation in Acute Ischemic Stroke: Hemorrhagic Subtypes and Symptomatic Intracranial Hemorrhage,” Journal of NeuroInterventional Surgery 17, no. 7 (2025): 673–682. 38969497 10.1136/jnis-2024-021725 PMC12322388

[43] C. H. Chen , A. Shoamanesh , P. Colorado , et al., “Hemorrhagic Infarction Does Not Worsen Functional Outcomes in Noncardioembolic Ischemic Stroke: Secondary Analysis From PACIFIC‐STROKE,” Stroke 56, no. 7 (2025): 1730–1737. 40197044 10.1161/STROKEAHA.124.049188

[44] J. G. Filep , A. Delalandre , Y. Payette , and E. Földes‐Filep , “Glucocorticoid Receptor Regulates Expression of L‐Selectin and CD11/CD18 on Human Neutrophils,” Circulation 96, no. 1 (1997): 295–301. 9236448 10.1161/01.cir.96.1.295

[45] K. De Bosscher , W. Vanden Berghe , L. Vermeulen , S. Plaisance , E. Boone , and G. Haegeman , “Glucocorticoids Repress NF‐kappaB‐Driven Genes by Disturbing the Interaction of p65 With the Basal Transcription Machinery, Irrespective of Coactivator Levels in the Cell,” Proceedings of the National Academy of Sciences of the United States of America 97, no. 8 (2000): 3919–3924. 10760263 10.1073/pnas.97.8.3919 PMC18117

[46] S. Yuan , K. J. Liu , and Z. Qi , “Occludin Regulation of Blood‐Brain Barrier and Potential Therapeutic Target in Ischemic Stroke,” Brain Circulation 6, no. 3 (2020): 152–162. 33210038 10.4103/bc.bc_29_20 PMC7646391

[47] H. Li , H. Wen , J. Liu , et al., “The Glycocalyx: A Key Target for Treatment of Severe Acute Pancreatitis‐Associated Multiple Organ Dysfunction Syndrome,” Human Cell 38, no. 4 (2025): 107. 40411704 10.1007/s13577-025-01227-6 PMC12103372

[48] K. H. Moore , H. A. Murphy , and E. M. George , “The Glycocalyx: A Central Regulator of Vascular Function,” American Journal of Physiology. Regulatory, Integrative and Comparative Physiology 320, no. 4 (2021): R508–R518. 33501896 10.1152/ajpregu.00340.2020 PMC8238147

[49] C. Dancy , K. E. Heintzelman , and M. E. Katt , “The Glycocalyx: The Importance of Sugar Coating the Blood‐Brain Barrier,” International Journal of Molecular Sciences 25, no. 15 (2024): 8404. 39125975 10.3390/ijms25158404 PMC11312458

[50] X. Xie , L. Wang , S. Dong , S. Ge , and T. Zhu , “Immune Regulation of the Gut‐Brain Axis and Lung‐Brain Axis Involved in Ischemic Stroke,” Neural Regeneration Research 19, no. 3 (2024): 519–528. 37721279 10.4103/1673-5374.380869 PMC10581566

[51] L. Mascia , R. D'Albo , I. Cavalli , L. Giaccari , M. Della Giovampaola , and B. Donati , “Organ Crosstalk: Brain‐Lung Interaction,” Frontiers in Medicine (Lausanne) 12 (2025): 1655813. 10.3389/fmed.2025.1655813 PMC12417159 40933561

[52] P. F. Dequin , F. Meziani , J. P. Quenot , et al., “Hydrocortisone in Severe Community‐Acquired Pneumonia,” New England Journal of Medicine 388, no. 21 (2023): 1931–1941. 36942789 10.1056/NEJMoa2215145

[53] J. de Gans , D. van de Beek , and Investigators EDiABMS , “Dexamethasone in Adults With Bacterial Meningitis,” New England Journal of Medicine 347, no. 20 (2002): 1549–1556. 12432041 10.1056/NEJMoa021334

