# Supplementary material for: Adjunctive Methylprednisolone After Thrombectomy: A Secondary Analysis Stratified by Admission White Blood Cell Count
Source: CNS Neurosci Ther. 2026 May 31;32(6):e70956. doi: 10.1002/cns.70956 (PMC13240513; doi:10.1002/cns.70956)
Supplement: Supplementary file 1 — Data S1: Supporting Information. [file CNS-32-e70956-s001.docx]

**Supplementary Appendix**

This Supplementary Appendix provides additional methodological details and supporting analyses for the post hoc secondary analysis of the MARVEL trial stratified by admission white blood cell count. It includes expanded statistical methods and model specifications, propensity-score matching procedures, definitions of analysis sets and covariates, multiple-imputation methods, subgroup-analysis methods, and the time-stamped statistical analysis plan for the present analysis. It also includes supplementary tables reporting interaction analyses, within-treatment WBC comparisons, propensity-score–matched analyses, LASSO-sensitivity analyses, and multiple-imputation sensitivity analyses, as well as supplementary figures showing covariate balance, LASSO diagnostics, missing-data patterns, multiple-imputation diagnostics, and patient-flow visualization.

**Contents**

[eMethods 2](#_Toc228887863)

[eMethods 1. Statistical details and model specification. 2](#_Toc228887864)

[eMethods 2. Propensity‑score matching (PSM) 4](#_Toc228887865)

[eMethods 3. Analysis Sets & Covariates 4](#_Toc228887866)

[eMethods 4. Multiple imputation (MI) 5](#_Toc228887867)

[eMethods 5. Subgroup Analyses 5](#_Toc228887868)

[eMethods 6**.** Time-stamped statistical analysis plan for the present analysis（October 15, 2024） 6](#_Toc228887869)

[eTables 10](#_Toc228887870)

[eTable 1. Treatment×WBC Interaction: Stratum-Specific Effects (MPSS vs Placebo) and Interaction P Values. 10](#_Toc228887871)

[eTable 2. Clinical Outcomes by WBC Level Within Each Treatment Group: High WBC (≥10×10⁹/L) Versus Low WBC (<10×10⁹/L) in the Placebo and MPSS Cohorts 14](#_Toc228887872)

[eTable 3. Propensity-Score–Matched Baseline Characteristics by Treatment Group Within Admission WBC Strata 19](#_Toc228887873)

[eTable 4. Clinical Outcomes by Treatment Within Admission WBC Strata: Propensity-Score–Matched Cohorts With Double Adjustment for Residual Imbalance 23](#_Toc228887874)

[eTable 5. Clinical Outcomes by Treatment Within Admission WBC Strata: LASSO-Sensitivity Analyses 27](#_Toc228887875)

[eTable 6. Clinical Outcomes by Treatment Within Admission WBC Strata: Multiple-Imputation Sensitivity Analyses 31](#_Toc228887876)

[Figures 35](#_Toc228887877)

[eFigure S1. Love Plots of Standardized Mean Differences Before and After Matching, by WBC Stratum 35](#_Toc228887878)

[eFigure S2. LASSO Variable Selection: Cross-Validation Error Curves and Coefficient Paths 37](#_Toc228887879)

[eFigure S3 Horizontal Dot Plot of Missing Observations per Variable. 40](#_Toc228887880)

[eFigure S4. Multiple-Imputation Diagnostics (MICE) 42](#_Toc228887881)

[eFigure S5. Sankey Diagram of Patient Flows by Baseline WBC Stratum, Treatment, and 90-Day mRS 45](#_Toc228887882)

# eMethods

## eMethods 1. Statistical details and model specification.

**Objective**: To assess whether the estimated effect of adjunctive methylprednisolone sodium succinate (MPSS) with endovascular thrombectomy (EVT) varied by admission white blood cell (WBC) level (<10 vs ≥10×10⁹/L).
**Populations**: From the MARVEL randomized population, we restricted the present post hoc secondary analysis to patients with successful reperfusion (eTICI ≥2b) and available admission WBC measured before randomization. The primary analysis cohort was the baseline-covariate complete-case primary cohort, defined by available admission WBC and complete adjustment covariates specified in the analysis plan. Clinical outcomes were analyzed as observed and were not imputed; therefore, model denominators could vary across endpoints according to outcome availability.
**Outcomes and coding**: The primary endpoint was the 90-day ordinal modified Rankin Scale (mRS 0–6). Secondary endpoints comprised: (a) functional outcomes—mRS 0–1, 0–2, 0–3, and 0–4; NIHSS at 5–7 days (lower is better); and EQ-5D-VAS at 90 days (higher is better); and (b) safety outcomes—all-cause mortality, symptomatic intracranial hemorrhage (sICH), any intracranial hemorrhage (ICH), pneumonia, and gastrointestinal bleeding. For functional endpoints, OR > 1 favors MPSS. For continuous outcomes, β < 0 favors MPSS for NIHSS, whereas β > 0 favors MPSS for EQ-5D-VAS. For adverse safety endpoints, OR < 1 favors MPSS.
**Models**: Proportional-odds cumulative logit models were used for the ordinal mRS outcome, with results reported as adjusted common odds ratios (aCORs). Binary endpoints used multivariable logistic regression, with results reported as adjusted odds ratios (aORs). Where reported, adjusted risk differences (aRDs) were estimated by marginal standardization from the fitted logistic models, with uncertainty quantified using robust sandwich standard errors. Continuous endpoints used linear regression, with results reported as adjusted mean differences (β).
Effect modification: Effect modification was assessed by fitting multivariable models that included treatment assignment, WBC stratum, and a treatment-by-WBC interaction term; interactions were tested using Wald tests (two-sided α = 0.05). Interaction analyses were conducted in three analysis sets: (i) the baseline-covariate complete-case primary cohort (n = 1201) using MARVEL-aligned covariates specified in the analysis plan; (ii) the same baseline-covariate complete-case cohort using LASSO-selected covariates as a sensitivity analysis; and (iii) a multiple-imputation (MI) sensitivity cohort, in which missing baseline covariates were imputed (m = 5) and outcomes were analyzed as observed. Admission WBC used for stratification was required and was not imputed. Corresponding interaction P values for the primary, LASSO-sensitivity, and MI-sensitivity analyses are provided in eTable 1.
Within-stratum treatment comparisons: Within each WBC stratum, treatment effects (MPSS vs placebo) were estimated using two covariate specifications: (a) MARVEL-aligned covariates specified in the analysis plan, used for the primary and MI-sensitivity analyses; and (b) LASSO-selected covariates, used for the LASSO-sensitivity analyses.
**Multiplicity**: Within each analysis set, Holm–Bonferroni adjustment was applied separately within each WBC stratum to the family of four dichotomized functional thresholds: mRS 0–1, 0–2, 0–3, and 0–4. The two WBC strata were not pooled for this multiplicity adjustment. The primary ordinal endpoint and safety endpoints were not multiplicity-adjusted. Reported P values are nominal unless otherwise specified.
**Software**: Analyses used R version 4.3.2 (glmnet, ordinal, MatchIt, mice, sandwich), with SPSS version 26.0 and GraphPad Prism version 8 for data management and graphics.

## eMethods 2. Propensity‑score matching (PSM)

Within each WBC stratum, propensity scores were estimated using logistic regression of treatment assignment on the MARVEL-aligned baseline covariates specified in the analysis plan. Patients were then matched 1:1 on the logit of the propensity score using nearest-neighbor matching with a caliper of 0.10, without replacement. Balance was assessed using standardized mean differences (SMDs), with |SMD| < 0.10 indicating adequate balance.
After matching, outcome models applied double adjustment for analysis-plan covariates that showed residual imbalance after matching (|SMD| > 0.10). Because the set of residual imbalances differed between the low-WBC and high-WBC strata, the additional adjustment covariates were not identical across strata, as detailed in eTable 4.

## eMethods 3. Analysis Sets & Covariates

**Candidate pool for LASSO-sensitivity analyses**: demographics; vascular risk factors/comorbidities; vital signs; laboratory indices, including white blood cell count (WBC) and lymphocyte count (LC); imaging grades/scores, including NIHSS, ASPECTS, and ASITN/SIR collateral grade; occlusion site; anesthesia; workflow times, including onset-to-puncture time (OTP) and puncture-to-reperfusion time (PTR); and other clinically relevant baseline variables.
Primary and MI-sensitivity analyses used the MARVEL-aligned covariate set specified in the analysis plan. This covariate set comprised age, prestroke mRS score, baseline NIHSS score, baseline ASPECTS, use of intravenous thrombolysis (IVT), onset-to-randomization time (OTR), and occlusion site.
LASSO runs were conducted for three analysis sets: (1) the overall cohort for interaction models; (2) the low-WBC stratum; and (3) the high-WBC stratum. The following final covariate lists refer to variables selected by LASSO for the LASSO-sensitivity analyses.
Final covariate lists used in treatment comparisons were as follows:

**(i) Overall cohort for interaction models**: age; white blood cell count (WBC); lymphocyte count (LC); glucose; baseline National Institutes of Health Stroke Scale (NIHSS) score; baseline Alberta Stroke Program Early CT Score (ASPECTS); American Society of Interventional and Therapeutic Neuroradiology/Society of Interventional Radiology (ASITN/SIR) collateral grade; Trial of Org 10172 in Acute Stroke Treatment (TOAST) subtype; occlusion site; general anesthesia; and puncture-to-reperfusion time (PTR).
**(ii) Low-WBC stratum**: age; hypertension; hyperlipidemia; lymphocyte count (LC); glucose; baseline NIHSS score; baseline ASPECTS; ASITN/SIR collateral grade; TOAST subtype; occlusion site; general anesthesia; onset-to-puncture time (OTP); and PTR.
**(iii) High-WBC stratum**: age; baseline NIHSS score; baseline ASPECTS; ASITN/SIR collateral grade; and PTR.

eMethods 4. Multiple imputation (MI)
Missing baseline covariate data were handled using multiple imputation by chained equations (MICE; m = 5; maxit = 20; seed = 2025). Imputation was applied only to missing baseline covariates: predictive mean matching was used for continuous variables, logistic models for binary variables, and cumulative-logit models for ordered variables. Admission WBC was required for stratification and was not imputed.
All outcome variables, including the 90-day mRS (0–6), mortality, symptomatic intracranial hemorrhage (sICH), any intracranial hemorrhage (ICH), pneumonia, gastrointestinal bleeding, NIHSS, and EQ-5D-VAS, were analyzed as observed and were not imputed. Therefore, outcome-specific model denominators reflected the number of patients with available data for each outcome. Vital status was not modeled separately. Imputation diagnostics, including missingness patterns, observed-versus-imputed distribution overlays, and iteration trace/convergence plots, are provided in the eFigures. Estimates were combined using Rubin’s rules.

eMethods 5. Subgroup Analyses
Subgroup analyses were conducted to assess the consistency of treatment estimates and to explore potential effect modification of adjunctive methylprednisolone after thrombectomy. The subgroup framework was specified in the analysis plan as supportive and nonconfirmatory, guided by biological plausibility and data-driven feature selection.
Variable selection.
Candidate subgroup variables were informed by a LASSO-based procedure specified before outcome modeling and supplemented by clinically relevant variables commonly reported in thrombectomy studies. Final variables included age, baseline NIHSS, baseline ASPECTS, serum glucose, occlusion site, puncture-to-reperfusion time (procedure time), collateral grade (ASITN/SIR), and etiologic subtype (TOAST). These variables were used to test potential interactions with treatment assignment (MPSS vs placebo).
**Interpretation.**
Subgroup findings are exploratory and should be interpreted as supportive evidence complementing the primary treatment×WBC interaction analysis.

eMethods 6. Time-stamped statistical analysis plan for the present analysis（October 15, 2024）

**Parent trial eligibility**. Participants were enrolled in the MARVEL randomized, double-blind trial per the parent protocol, with eligibility criteria, treatment windows, and contraindications defined therein. All trial screening, randomization, treatment allocation, and outcome assessment followed the MARVEL protocol and were adjudicated at participating sites.
**Secondary-analysis cohort for the present study.** From the MARVEL intention-to-treat population, the analysis cohort was constructed using the following rules specified in this analysis plan.
**Inclusion criteria for this analysis:**
(1) Symptomatic anterior-circulation large-vessel occlusion enrolled in MARVEL.
(2) Successful reperfusion after thrombectomy, defined as eTICI ≥2b.
(3) Admission WBC measured before EVT/randomization and available for stratification.
**Exclusion criteria for this analysis:**
(1) Unsuccessful reperfusion, defined as eTICI <2b.
(2) Missing admission WBC.
(3) For the baseline-covariate complete-case primary cohort, missing any adjustment covariates specified in this analysis plan: age, prestroke mRS, baseline NIHSS, baseline ASPECTS, IVT use, onset-to-randomization time (OTR), or occlusion site.
Patients with missing outcome data were excluded only from the corresponding outcome-specific models. Outcomes were analyzed as observed and were not imputed.
**Rationale and objective.** This secondary analysis was not prespecified in the original trial protocol but followed a statistical analysis plan finalized and time-stamped before outcome modeling. The objective was to assess whether admission white blood cell (WBC) count modified the estimated effect of adjunctive methylprednisolone sodium succinate (MPSS) in patients undergoing EVT, with the primary modifier dichotomized as WBC <10 versus ≥10×10⁹/L.
**Analysis populations**.
Primary analysis set: baseline-covariate complete-case primary cohort, defined by available admission WBC and complete adjustment covariates specified in this analysis plan.
MI-sensitivity analysis set: multiply imputed dataset retaining patients who otherwise met the eligibility criteria for this analysis but had one or more missing baseline covariates. Missing baseline covariates were imputed; outcomes were analyzed as observed and were not imputed.
**Endpoints and directionality.**
Primary endpoint: 90-day ordinal mRS (0–6).
Secondary functional endpoints: mRS 0–1, 0–2, 0–3, and 0–4; NIHSS at days 5–7, where lower values are better; and EQ-5D-VAS at day 90, where higher values are better.
Safety endpoints: all-cause mortality, symptomatic intracranial hemorrhage (sICH), any intracranial hemorrhage (ICH), pneumonia, and gastrointestinal bleeding.
**Directionality:** For ordinal mRS, aCOR > 1 favors MPSS. For functional binary endpoints, aOR > 1 favors MPSS. For adverse safety endpoints, aOR < 1 favors MPSS. For continuous endpoints, β < 0 favors MPSS for NIHSS, whereas β > 0 favors MPSS for EQ-5D-VAS.
**Estimands and models.**
Ordinal mRS: proportional-odds cumulative-logit model, reporting adjusted common odds ratio (aCOR).
Binary endpoints: multivariable logistic regression, reporting adjusted odds ratio (aOR); where specified, adjusted risk difference (aRD) estimated from fitted models with robust standard errors.
Continuous endpoints: linear regression, reporting β coefficients.
Fixed terms in interaction models included treatment assignment, WBC stratum, and the treatment×WBC interaction term. Interaction tests were two-sided with α = 0.05.
**Covariates specified in this analysis plan.**
Primary and MI-sensitivity analyses adjusted for the MARVEL RCT–aligned covariate set: age, prestroke mRS, baseline NIHSS, baseline ASPECTS, IVT use, onset-to-randomization time (OTR), and occlusion site.

**Missing data.**
Multiple imputation by chained equations was applied to missing baseline covariates only (m = 5; maxit = 20; seed = 2025), using predictive mean matching for continuous variables, logistic models for binary variables, and cumulative-logit models for ordered variables. Admission WBC was required for stratification and was not imputed. All outcomes, including 90-day mRS, mortality, sICH, any ICH, pneumonia, gastrointestinal bleeding, NIHSS, and EQ-5D-VAS, were analyzed as observed and were not imputed. Therefore, outcome-specific model denominators varied according to outcome availability. Estimates were combined using Rubin’s rules.
**Multiplicity.**Holm–Bonferroni adjustment was applied separately within each WBC stratum to the family of four dichotomized functional thresholds: mRS 0–1, 0–2, 0–3, and 0–4. The two WBC strata were not pooled for this multiplicity adjustment. The primary ordinal endpoint and safety endpoints were not multiplicity-adjusted. Reported *P* values are nominal unless otherwise specified.
**Sensitivity analyses.**
Multiple imputation (MI). Missing baseline covariates were imputed using MICE (m = 5; maxit = 20; seed = 2025), as described above. All outcomes, including the 90-day mRS (0–6), mortality, sICH, any ICH, pneumonia, gastrointestinal bleeding, NIHSS, and EQ-5D-VAS, were analyzed as observed with no imputation. The same MARVEL-aligned covariate set specified in this analysis plan was used; estimates were combined using Rubin’s rules.
Propensity-score matching (PSM) within WBC strata. Patients were matched 1:1 within each WBC stratum using nearest-neighbor matching on the logit of the propensity score with a caliper of 0.10, without replacement. Balance was assessed using standardized mean differences (SMDs), with |SMD| < 0.10 indicating adequate balance. Double adjustment was applied for analysis-plan covariates that showed residual imbalance after matching (|SMD| > 0.10), and the additional adjustment set could therefore differ between the low-WBC and high-WBC strata.
Supportive LASSO-informed analyses. A LASSO-based procedure specified before outcome modeling was applied to baseline characteristics and supplemented by commonly used clinical variables to inform the choice of subgroup variables for interaction testing, both overall and within the low-WBC and high-WBC strata. For each subgroup variable, a treatment×subgroup interaction term was fitted in the proportional-odds model. These results were nonconfirmatory and did not replace inference from the primary model using the MARVEL-aligned covariate set. *P* values were interpreted as nominal without multiplicity adjustment.
High-WBC subgroup analyses. Within the high-WBC stratum (WBC ≥10×10⁹/L), the 90-day ordinal mRS (0–6) was modeled using a proportional-odds cumulative-logit model to estimate the MPSS versus placebo effect, reported as aCOR with 95% CI. Subgroup variables were selected using the LASSO-informed procedure described above, supplemented by commonly used clinical variables, including age, prestroke mRS, baseline NIHSS, baseline ASPECTS, IVT use, onset-to-randomization time, occlusion site, serum glucose, procedural time, collateral grade (ASITN/SIR), and etiologic subtype. For each subgroup, the treatment×subgroup interaction was tested within the proportional-odds model. These analyses were supportive and nonconfirmatory; *P* values were nominal, with no multiplicity adjustment.
Study eligibility and sample construction. Participants were enrolled in the MARVEL randomized, double-blind trial per the parent protocol. For the present post hoc secondary analysis, the cohort was constructed using the eligibility and exclusion criteria described above. The baseline-covariate complete-case primary cohort included patients with available admission WBC and complete adjustment covariates specified in this analysis plan. Patients with missing outcome data contributed to models for outcomes that were available and were excluded only from models for outcomes that were missing.
The MI-sensitivity set retained patients who otherwise met the eligibility criteria for this analysis but had one or more missing baseline covariates; outcomes were not imputed.
Sample construction and numbers at each step are detailed in Figure 1 and correspond to the denominators reported in the main tables and supplementary tables.
**Timeline.** The statistical analysis plan for this post hoc secondary analysis was finalized and time-stamped on October 15, 2024, before outcome modeling or interaction testing. All analyses were conducted on the locked MARVEL dataset.

# eTables

## eTable 1. Treatment×WBC Interaction: Stratum-Specific Effects (MPSS vs Placebo) and Interaction P Values.

|  | | | Adjusted effect (95% CI) | P value | P for interaction (primary) | P for interaction (LASSO-sensitivity) | P for interaction (MI-sensitivity) |  |
| --- | --- | --- | --- | --- | --- | --- | --- | --- |
| Primary efficacy outcome | | | |  |  |  |  |  |
|  | mRS score at 90 days | | |  |  |  |  |  |
|  |  | WBC<10 | aCOR: 0.96 (0.75 to 1.23) | 0.75 | 0.04 | 0.03 | 0.046 |  |
|  |  | WBC≥10 | aCOR: 1.59 (1.11 to 2.28) | 0.01 |  |  |  |  |
| Secondary efficacy outcomes | | | |  |  |  |  |  |
|  | mRS score of 0-1 at 90 days | | |  |  |  |  |  |
|  |  | WBC<10 | aOR: 0.91 (0.66 to 1.27) | 0.58 | 0.44† | 0.50† | 0.54† |  |
|  |  | WBC≥10 | aOR: 1.12 (0.67 to 1.85) | 0.67 |  |  |  |  |
|  | mRS score of 0-2 at 90 days | | |  |  |  |  |  |
|  |  | WBC<10 | aOR: 1.01 (0.74 to 1.37) | 0.96 | 0.12† | 0.17† | 0.21† |  |
|  |  | WBC≥10 | aOR: 1.54 (0.98 to 2.41) | 0.06 |  |  |  |  |
|  | mRS score of 0-3 at 90 days | | |  |  |  |  |  |
|  |  | WBC<10 | aOR: 1.01 (0.73 to 1.39) | 0.97 | 0.05† | 0.04† | 0.15† |  |
|  |  | WBC≥10 | aOR: 1.86 (1.19 to 2.92) | 0.01 |  |  |  |  |
|  | mRS score of 0-4 at 90 days | | |  |  |  |  |  |
|  |  | WBC<10 | aOR: 1.09 (0.77 to 1.55) | 0.63 | 0.13† | 0.10† | 0.16† |  |
|  |  | WBC≥10 | aOR: 1.82 (1.15 to 2.87) | 0.01 |  |  |  |  |
|  | NIHSS score at 5-7 days | | |  |  |  |  |  |
|  |  | WBC<10 | β: 0.28 (-1.36 to 1.92) | 0.74 | 0.14 | 0.21 | 0.30 |  |
|  |  | WBC≥10 | β: -2.03 (-4.40 to 0.35) | 0.10 |  |  |  |  |
|  | EQ-5D-VAS score at 90 days | | | |  |  |  |  |
|  |  | WBC<10 | β: -0.65 (-5.14 to 3.84) | 0.78 | 0.05 | 0.06 | 0.12 |  |
|  |  | WBC≥10 | β: 8.06 (1.38 to 14.75) | 0.02 |  |  |  |  |
| Safety outcomes | | |  |  |  |  |  |  |
|  | Mortality | |  |  |  |  |  |  |
|  |  | WBC<10 | aOR: 0.91 (0.62 to 1.32) | 0.61 | 0.30 | 0.20 | 0.18 |  |
|  |  | WBC≥10 | aOR: 0.60 (0.37 to 0.96) | 0.03 |  |  |  |  |
|  | Symptomatic intracranial hemorrhage | | |  |  |  |  |  |
|  |  | WBC<10 | aOR: 0.89 (0.53 to 1.50) | 0.67 | 0.70 | 0.91 | 0.77 |  |
|  |  | WBC≥10 | aOR: 0.81 (0.44 to 1.48) | 0.49 |  |  |  |  |
|  | Any radiologic intracranial hemorrhage | | | |  |  |  |  |
|  |  | WBC<10 | aOR: 0.99 (0.73 to 1.34) | 0.94 | 0.08 | 0.15 | 0.28 |  |
|  |  | WBC≥10 | aOR: 1.41 (0.93 to 2.10) | 0.10 |  |  |  |  |
|  | Gastrointestinal bleeding within 7 days after EVT | | | |  |  |  |  |
|  |  | WBC<10 | aOR: 0.51 (0.24 to 1.08) | 0.08 | 0.49 | 0.58 | 0.31 |  |
|  |  | WBC≥10 | aOR: 0.64 (0.30 to 1.36) | 0.25 |  |  |  |  |
|  | Pneumonia | | |  |  |  |  |  |
|  |  | WBC<10 | aOR: 0.81 (0.60 to 1.08) | 0.15 | 0.47 | 0.28 | 0.64 |  |
|  |  | WBC≥10 | aOR: 0.61 (0.40 to 0.93) | 0.02 |  |  |  |  |
| Notes. Interaction tests were evaluated in the baseline-covariate complete-case primary cohort (n = 1201), the LASSO-sensitivity analysis using LASSO-selected covariates, and the multiple-imputation (MI) sensitivity analysis. The primary models used the MARVEL-aligned covariate set specified in the analysis plan; LASSO-sensitivity models used the covariate sets described in eMethods 3. Baseline WBC strata were defined as <10 versus ≥10×10⁹/L.  For each outcome, the adjusted effect estimate represents the treatment effect of MPSS versus placebo within the corresponding WBC stratum. Interaction models included treatment assignment, WBC stratum, and the treatment×WBC interaction term. Blank cells in the interaction columns indicate that the interaction *P* value applies to the corresponding outcome-level interaction test and is shown once for each outcome.  Ordinal mRS (0–6) models were parameterized so that aCOR > 1 indicates a shift toward better outcomes, that is, lower mRS scores, with MPSS versus placebo. For functional thresholds (mRS 0–1, 0–2, 0–3, and 0–4), events were coded as achieving the threshold; therefore, aOR > 1 favors MPSS. For safety endpoints, including mortality, sICH, any ICH, gastrointestinal bleeding, and pneumonia, events were coded as occurrences; therefore, aOR < 1 favors MPSS. For continuous endpoints, β < 0 favors MPSS for NIHSS, where lower scores are better, and β > 0 favors MPSS for EQ-5D-VAS, where higher scores are better.  Holm–Bonferroni adjustment was applied separately within each WBC stratum to the family of four dichotomized functional thresholds: mRS 0–1, 0–2, 0–3, and 0–4. The two WBC strata were not pooled for this multiplicity adjustment. The primary ordinal endpoint, safety endpoints, continuous endpoints, and interaction tests were not multiplicity-adjusted. Reported *P* values are nominal unless otherwise specified.  Abbreviations: aCOR, adjusted common odds ratio; aOR, adjusted odds ratio; CI, confidence interval; EQ-5D-VAS, EuroQol 5-Dimension visual analog scale; ICH, intracranial hemorrhage; MI, multiple imputation; MPSS, methylprednisolone sodium succinate; mRS, modified Rankin Scale; NIHSS, National Institutes of Health Stroke Scale; sICH, symptomatic intracranial hemorrhage; WBC, white blood cell count. | | | | | | | |  |

## eTable 2. Clinical Outcomes by WBC Level Within Each Treatment Group: High WBC (≥10×10⁹/L) Versus Low WBC (<10×10⁹/L) in the Placebo and MPSS Cohorts

|  | | No./total (%) | | | | No./total (%) | | | |
| --- | --- | --- | --- | --- | --- | --- | --- | --- | --- |
|  | | Placebo stratum (n=597) | | | | MPSS stratum (n=604) | | | |
|  | | Low-WBC group (n=407) | High-WBC group (n=190) | Adjusted effect (95% CI) | *P* value | Low-WBC group (n=401) | High-WBC group (n=203) | Adjusted effect (95% CI) | *P* value |
| Primary efficacy outcome *a* | | |  |  |  |  |  |  |  |
| mRS score at 90 days, median (IQR) | | 3 (1 to 5) | 4 (2 to 6) | aCOR: 0.55 (0.40 to 0.76) | <0.001 | 3.0 (1.0 to 5.0) | 3.0 (1.8 to 5.0) | aCOR: 0.87 (0.64 to 1.18) | 0.37 |
| Secondary efficacy outcomes *b* | | |  |  |  |  |  |  |  |
|  | mRS score of 0-1 at 90 days | 125/404 (30.9) | 44/190 (23.2) | aOR: 0.61 (0.39 to 0.94) | 0.02† | 120/399 (30.1) | 50/202 (24.8) | aOR: 0.71 (0.47 to 1.08) | 0.11† |
|  |  |  |  | aRD: -10.06 (-17.82 to -2.30) | 0.01 |  |  | aRD: -6.27 (-14.05 to 1.51) | 0.11 |
|  | mRS score of 0-2 at 90 days | 190/404 (47.0) | 69/190 (36.3) | aOR: 0.60 (.40 to .89) | 0.01† | 192/399 (48.1) | 91/202 (45.0) | aOR: 0.83 (0.57 to 1.21) | 0.34† |
|  |  |  |  | aRD: -13.42 (-22.89 to -3.96) | 0.01 |  |  | aRD: -4.69 (-13.92 to 4.54) | 0.32 |
|  | mRS score of 0-3 at 90 days | 249/404 (61.6) | 92/190 (48.4) | aOR: 0.46 (0.31 to 0.70) | <0.001† | 250/399 (62.7) | 124/202 (61.4) | aOR: 0.86 (0.58 to 1.27) | 0.45† |
|  |  |  |  | aRD: -18.45 (-28.25 to -8.67) | <0.001 |  |  | aRD: -3.31 (-12.02 to 5.40) | 0.46 |
|  | mRS score of 0-4 at 90 days | 290/404 (71.8) | 114/190 (60.0) | aOR: 0.50 (0.33 to 0.75) | 0.001† | 293/399 (73.4) | 146/202 (72.3) | aOR: 0.86 (0.56 to 1.31) | 0.48† |
|  |  |  |  | aRD: -14.27 (-23.01 to -5.52) | 0.001 |  |  | aRD: -2.80 (-10.21 to 4.60) | 0.46 |
|  | NIHSS score at 5–7 days, median (IQR) | 9.5 (3.0 to 19.0) | 13.0 (6.0 to 31.0) | β: 3.89 (1.79 to 5.98) | <0.001 | 10.0 (2.0 to 21.0) | 11.0 (4.98 to 23.0) | β: 1.81 (-.18 to 3.80) | 0.08 |
|  | EQ-5D-VAS score at 90 days, median (IQR) | 60 (0 to 80) | 30 (0 to 80) | β: -10.69 (-16.46 to -4.92) | <0.001 | 55 (5 to 85) | 55 (5 to 80) | β: -2.27 (-7.77 to 3.24) | 0.42 |
| Safety outcomes | | |  |  |  |  |  |  |  |
|  | Mortality | 92/404 (22.8) | 63/190 (33.2) | aOR: 1.98 (1.29 to 3.05) | 0.002 | 85/399 (21.3) | 47/202 (23.3) | aOR: 1.21 (0.77 to 1.89) | 0.41 |
|  |  |  |  | aRD: 11.66 (3.78 to 19.55) | 0.004 |  |  | aRD: 2.96 (-3.92 to 9.83) | 0.40 |
|  | sICH | 35/403 (8.7) | 28/189 (14.8) | aOR: 1.75 (1.01 to 3.05) | 0.05 | 31/393 (7.9) | 25/202 (12.4) | aOR: 1.80 (1.00 to 3.25) | 0.05 |
|  |  |  |  | aRD: 5.06 (-0.43 to 10.55) | 0.07 |  |  | aRD: 3.90 (-0.60 to 8.39) | 0.09 |
|  | Any radiologic intracranial hemorrhage | 137/403 (34.0) | 81/189 (42.9) | aOR: 1.41 (.98 to 2.02) | 0.07 | 133/393 (33.8) | 101/202 (50.0) | aOR: 2.07 (1.44 to 2.96) | <0.001 |
|  |  |  |  | aRD:8.07 (-0.52 to 16.66) | 0.07 |  |  | aRD:16.86 (8.30 to 25.43) | <0.001 |
|  | Pneumonia | 197/407 (48.4) | 116/190 (61.1) | aOR: 1.91 (1.30 to 2.79) | 0.001 | 170/401 (42.4) | 101/203 (49.8) | aOR: 1.39 (0.97 to 1.98) | 0.07 |
|  |  |  |  | aRD:15.76 (6.77 to 24.74) | <0.001 |  |  | aRD:8.09 (-0.67 to 16.85) | 0.07 |
|  | Gastrointestinal bleeding within 7 days after EVT | 21/407 (5.2) | 18/190 (9.5) | aOR: 1.76 (.89 to 3.48) | 0.10 | 12/401 (3.0) | 13/203 (6.4) | aOR: 2.56 (1.11 to 5.92) | 0.03 |
|  |  |  |  | aRD:2.94 (-1.08 to 6.97) | 0.15 |  |  | aRD:3.81 (-0.2 to 7.8) | 0.06 |
| **Design rationale.** This table presents a complementary analysis to eTable 1. Instead of comparing MPSS versus placebo within each WBC stratum, as in Table 2 and eTable 1, this analysis compares high versus low WBC within each treatment cohort separately. This within-treatment perspective helps contextualize the treatment×WBC interaction: in the placebo cohort, high WBC tended to be associated with worse outcomes, whereas in the MPSS cohort this adverse WBC gradient appeared attenuated.  Data are presented as No. (%) unless otherwise indicated. Denominators may vary because of missing outcome data. Admission WBC strata were defined as <10 versus ≥10×10⁹/L.  **Design of this table.** Outcomes compare high WBC (≥10×10⁹/L) versus low WBC (<10×10⁹/L) within each treatment cohort separately: placebo and MPSS. Effect estimates were adjusted within each treatment cohort.  **Model specification and interpretation.** – Ordinal endpoint (mRS 0–6): proportional-odds models were used to estimate aCORs for high versus low WBC. An aCOR <1 indicates a shift toward worse outcomes, that is, higher mRS scores, in the high-WBC group. – Functional thresholds (mRS 0–1, 0–2, 0–3, and 0–4): multivariable logistic regression was used to estimate aORs for high versus low WBC. An aOR <1 indicates a lower probability of achieving the functional threshold in the high-WBC group. – Safety endpoints, including mortality, sICH, any ICH, gastrointestinal bleeding, and pneumonia: logistic regression was used to estimate aORs for high versus low WBC. An aOR >1 indicates a higher risk in the high-WBC group. – Continuous endpoints: linear models were used to estimate adjusted mean differences, calculated as high WBC minus low WBC. For NIHSS at 5–7 days, positive values indicate worse scores; for EQ-5D-VAS at 90 days, positive values indicate better scores.  **Adjusted covariates.** All models were adjusted for the MARVEL-aligned covariate set specified in the analysis plan: age, prestroke mRS score, baseline NIHSS score, baseline ASPECTS, use of intravenous thrombolysis, onset-to-randomization time, and occlusion site.  **Multiplicity.** Within each treatment cohort, Holm–Bonferroni adjustment was applied to the family of four dichotomized functional thresholds: mRS 0–1, 0–2, 0–3, and 0–4. The placebo and MPSS cohorts were not pooled for this multiplicity adjustment. Reported table *P* values are nominal unless otherwise specified. Holm-adjusted *P* values were as follows: placebo cohort, mRS 0–1, *P* = 0.02; mRS 0–2, *P* = 0.02; mRS 0–3, *P* < 0.001; and mRS 0–4, *P* = 0.004. MPSS cohort, mRS 0–1, *P* = 0.44; mRS 0–2, *P* = 1.00; mRS 0–3, *P* = 1.00; and mRS 0–4, *P* = 1.00.  **Abbreviations:** aCOR, adjusted common odds ratio; aOR, adjusted odds ratio; aRD, adjusted risk difference; CI, confidence interval; EQ-5D-VAS, EuroQol 5-Dimension visual analog scale; ICH, intracranial hemorrhage; IQR, interquartile range; MPSS, methylprednisolone sodium succinate; mRS, modified Rankin Scale; NIHSS, National Institutes of Health Stroke Scale; sICH, symptomatic intracranial hemorrhage; WBC, white blood cell. | | | | | | | | | |

## eTable 3. Propensity-Score–Matched Baseline Characteristics by Treatment Group Within Admission WBC Strata

| Variables | | Low-WBC stratum (<10×10⁹/L, n=692) | | |  | High-WBC stratum (≥10×10⁹/L, n=324) | | |
| --- | --- | --- | --- | --- | --- | --- | --- | --- |
|  |  | Placebo group (n=346) | MPSS group (n=346) | SMD |  | Placebo group (n=162) | MPSS group (n=162) | SMD |
| Age, y, median (IQR) | | 70.0 (59.0–78.0) | 70.0 (59.0–76.0) | 0.07 |  | 65.5 (56.0–74.0) | 66.0 (57.0–73.0) | 0.05 |
| Sex, male, n (%) | | 192 (55.5) | 190 (54.9) | 0.01 |  | 104 (64.2) | 102 (63.0) | 0.03 |
| Glucose, mmol/L, median (IQR) | | 6.84 (5.90– 8.18) | 7.18 (6.10–8.55) | 0.03 |  | 7.40 (6.28–9.31) | 7.30 (6.31–9.06) | 0.03 |
| WBC, ×10⁹/L, median [IQR] | | 7.47 (6.31–8.73) | 7.44 (6.19–8.68) | 0.07 |  | 11.98 (10.87–13.81) | 12.38 (10.98–14.08) | 0.08 |
| LC, ×10⁹/L, median (IQR) | | 1.24 (0.83–1.73) | 1.26 (0.85–1.84) | 0.04 |  | 1.20 (0.90–1.68) | 1.16 (0.82–1.72) | 0.03 |
| BP, mmHg, median (IQR) | | |  |  |  |  |  |  |
|  | Systolic | 144.0 (127.0–159.0) | 144.0 (130.0–161.5) | 0.07 |  | 145.0 (126.0–158.75) | 143.0 (125.0–161.75) | 0.06 |
|  | Diastolic | 83.0 (74.0–93.0) | 82.5 (74.0–93.75) | 0.02 |  | 86.0 (74.0–95.75) | 85.0 (74.0–93.0) | 0.03 |
| Medical history, n (%) | | |  |  |  |  |  |  |
|  | Hypertension | 209 (60.4) | 200 (57.8) | 0.05 |  | 109 (67.3) | 107 (66.0) | 0.03 |
|  | Hyperlipidemia | 92 (26.6) | 106 (30.6) | 0.09 |  | 68 (42.0) | 61 (37.7) | 0.09 |
|  | Diabetes | 57 (16.5) | 65 (18.8) | 0.06 |  | 31 (19.1) | 38 (23.5) | **0.11** |
|  | Smoking | 94 (27.2) | 98 (28.3) | 0.03 |  | 48 (29.6) | 53 (32.7) | 0.07 |
|  | Atrial fibrillation | 166 (48.0) | 142 (41.0) | **0.14** |  | 53 (32.7) | 53 (32.7) | 0.00 |
|  | Coronary heart disease | 72 (20.8) | 73 (21.1) | 0.01 |  | 18 (11.1) | 20 (12.3) | 0.04 |
|  | Valvular heart disease | 55 (15.9) | 50 (14.5) | 0.04 |  | 17 (10.5) | 13 (8.0) | 0.09 |
| Prestroke mRS, n (%) | |  |  | 0.03 |  |  |  | 0.04 |
|  | 0 | 340 (98.3) | 339 (98.0) |  |  | 158 (97.5) | 159 (98.1) |  |
|  | 1 | 4 (1.2) | 5 (1.4) |  |  | 4 (2.5) | 3 (1.9) |  |
|  | 2 | 2 (0.6) | 2 (0.6) |  |  | 0 (0.0) | 0 (0.0) |  |
| ASPECTS, median (IQR) | | 6.0 (4.0–8.0) | 5.5 (4.0–7.0) | 0.08 |  | 5.0 (4.0–7.0) | 6.0 (4.0–7.0) | **0.11** |
| NIHSS score, median (IQR) | | 19.0 (16.0–21.0) | 19.0 (16.0–21.0) | 0.04 |  | 19.0 (17.0–21.0) | 18.5 (17.0–21.0) | 0.06 |
| ASITN/SIR collateral grade, n (%) | | |  | 0.02 |  |  |  | **0.14** |
|  | 0–1 | 129 (37.3) | 131 (37.9) |  |  | 67 (41.4) | 78 (48.1) |  |
|  | 2 | 126 (36.4) | 123 (35.5) |  |  | 47 (29.0) | 43 (26.5) |  |
|  | 3-4 | 91 (26.3) | 92 (26.6) |  |  | 48 (29.6) | 41 (25.3) |  |
| TOAST, n (%) | |  |  | 0.06 |  |  |  | **0.13** |
|  | LAA | 127 (36.7) | 122 (35.0) |  |  | 75 (46.3) | 66 (40.7) |  |
|  | CE | 182 (52.6) | 180 (52.0) |  |  | 64 (39.5) | 67 (41.4) |  |
|  | Others or undetermined | 37 (10.7) | 44 (12.7) |  |  | 23 (11.7) | 20 (12.3) |  |
| Occlusion site, n (%) | | |  | 0.03 |  |  |  | 0.05 |
|  | internal carotid | 121 (35.0) | 121 (35.0) |  |  | 58 (35.8) | 61 (37.7) |  |
|  | M1 segment | 190 (54.9) | 187 (54.0) |  |  | 85 (52.5) | 81 (50.0) |  |
|  | M2 segment | 35 (10.1) | 38 (11.0) |  |  | 19 (11.7) | 20 (12.3) |  |
| IVT, n (%) | | 135 (39.0) | 119 (34.4) | 0.10 |  | 59 (36.4) | 57 (35.2) | 0.03 |
| General anesthesia, n (%) | | 238 (68.8) | 241 (69.7) | 0.02 |  | 117 (72.2) | 111 (68.5) | 0.08 |
| OTR, min, median (IQR) | | 348.5(227.3–570.5) | 349.0 (227.3–632.5) | 0.03 |  | 387.0 (294.8–630.5) | 378.0 (255.5–618.0) | 0.02 |
| OTP, min, median (IQR) | | 344.0 (222.8–574.8) | 344.5 (235.0–637.0) | 0.03 |  | 394.5 (295.5–621.5) | 349.5 (246.3–568.8) | 0.01 |
| PTR, min, median (IQR) | | 63.0 (40.0–110.0) | 65.0 (39.0–105.0) | 0.04 |  | 70.0 (40.25–101.50) | 64.5 (38.0–113.5) | 0.03 |
| **Abbreviations:** ASITN/SIR, American Society of Interventional and Therapeutic Neuroradiology/Society of Interventional Radiology collateral grade; ASPECTS, Alberta Stroke Program Early CT Score; BP, blood pressure; CE, cardioembolism; DBP, diastolic blood pressure; EVT, endovascular thrombectomy; ICA, internal carotid artery; IQR, interquartile range; IVT, intravenous thrombolysis; LAA, large-artery atherosclerosis; LC, lymphocyte count; MPSS, methylprednisolone sodium succinate; mRS, modified Rankin Scale; NIHSS, National Institutes of Health Stroke Scale; OTR, onset-to-randomization time; OTP, onset-to-puncture time; PTR, puncture-to-reperfusion time; SBP, systolic blood pressure; SMD, standardized mean difference; WBC, white blood cell count.  Data are presented as median (IQR) or No. (%). Values are shown for each WBC stratum after 1:1 propensity-score matching of placebo and MPSS groups using nearest-neighbor matching on the logit of the propensity score with a caliper of 0.10, without replacement, as described in eMethods 2. One SMD per variable is reported; for categorical variables, the SMD summarizes imbalance across all levels jointly. All SMDs are reported to two decimal places. An absolute SMD <0.10 was considered to indicate adequate balance.  Most covariates achieved adequate balance after matching. Residual imbalances were observed for atrial fibrillation in the low-WBC stratum (SMD = 0.14) and for diabetes (SMD = 0.11), ASITN/SIR collateral grade (SMD = 0.14), ASPECTS (SMD = 0.11), and TOAST subtype (SMD = 0.13) in the high-WBC stratum; general anesthesia showed borderline imbalance (SMD = 0.08). These residual imbalances were accounted for by double adjustment in the propensity-score–matched outcome models. | | | | | | | | |

##

## eTable 4. Clinical Outcomes by Treatment Within Admission WBC Strata: Propensity-Score–Matched Cohorts With Double Adjustment for Residual Imbalance

|  | | **No./total (%)** | | | | **No./total (%)** | | | |
| --- | --- | --- | --- | --- | --- | --- | --- | --- | --- |
|  | | **Low-WBC stratum (<10×10⁹/L, n=692)** | | | | **High-WBC stratum (≥10×10⁹/L, n=324)** | | | |
|  | | Placebo group (n=346) | MPSS group (n=346) | Adjusted value  (95% CI) | *P value* | Placebo group (n=162) | MPSS group (n=162) | Adjusted value  (95% CI) | *P value* |
| **Primary efficacy outcome** *a* | | |  |  |  |  |  |  |  |
| mRS score at 90 days, median (IQR) | | 3 (1–5) | 3 (1–4) | aCOR: 1.16 (0.89 to 1.52) | 0.28 | 4.0 (1.5–6.0) | 3.0 (1.0–5.0) | aCOR: 1.50 (1.00 to 2.23) | .048 |
| **Secondary efficacy outcomes** *b* | | |  |  |  |  |  |  |  |
|  | mRS score of 0-1 at 90 days | 100/343 (29.2) | 105/344 (30.5) | aOR: 1.03 (0.72 to 1.45) | 0.87 | 40/162 (24.7) | 41/162 (25.3) | aOR: 1.05 (0.60 to 1.84) | 0.88 |
|  | mRS score of 0-2 at 90 days | 152/343 (44.3) | 169/344 (49.1) | aOR: 1.21 (0.87 to 1.69) | 0.27 | 62/162 (38.3) | 73/162 (45.1) | aOR: 1.37 (0.83 to 2.26) | 0.23 |
|  | mRS score of 0-3 at 90 days | 204/343 (59.5) | 221/344 (64.2) | aOR: 1.22 (0.86 to 1.74) | 0.27 | 78/162 (48.1) | 100/162 (61.7) | aOR: 2.00 (1.20 to 3.33) | 0.01 |
|  | mRS score of 0-4 at 90 days | 239/343 (69.7) | 259/344 (75.3) | aOR: 1.40 (0.95 to 2.06) | 0.09 | 99/162 (61.1) | 117/162 (72.2) | aOR: 1.83 (1.09 to 3.08) | 0.02 |
|  | NIHSS score at 5–7 days, median (IQR) | 10.0 (3.0–20.0) | 10.0 (2.0–19.0) | β: -0.67 (-2.40 to 1.05) | 0.44 | 13 (6, 31) | 10.5 (4.0–20.3) | β: -2.58 (-5.15 to -0.02) | 0.05 |
|  | EQ-5D-VAS score at 90 days, median (IQR) | 55 (0–80) | 60 (15–85) | β: 2.75 (-1.99 to 7.50) | 0.26 | 30 (0, 80) | 55 (0–75) | β: 6.80 (-0.30 to 13.91) | 0.06 |
| **Safety outcomes** | | |  |  |  |  |  |  |  |
|  | Mortality | 85/343 (24.8) | 68/344 (19.8) | aOR: 0.70 (0.47 to 1.06) | 0.09 | 50/162 (30.9) | 36/162 (22.2) | aOR: 0.57 (0.33 to 0.99) | 0.05 |
|  | Symptomatic intracranial hemorrhage | 30/342 (8.8) | 25/340 (7.4) | aOR: 0.81 (0.46 to 1.43) | 0.47 | 23/162 (14.2) | 18/162 (11.2) | aOR: 0.65 (0.31 to 1.33) | 0.65 |
|  | Any radiologic intracranial hemorrhage | 119/342 (34.8) | 113/340 (33.2) | aOR: 0.94 (0.68 to 1.29) | 0.68 | 70/162 (43.2) | 75/162 (46.6) | aOR: 1.06 (0.66 to 1.71) | 0.81 |
|  | Pneumonia | 172/346 (49.7) | 155/346 (43.6) | aOR: 0.81 (0.59 to 1.11) | 0.18 | 99/162 (61.1) | 76/162 (46.9) | aOR: 0.54 (0.34 to 0.86) | 0.01 |
|  | Gastrointestinal bleeding within 7 days after EVT | 20/346 (5.8) | 10/346 (2.9) | aOR: 0.46 (0.21 to 1.03) | 0.06 | 15/162 (9.3) | 9/162 (5.6) | aOR: 0.51 (0.20 to 1.30) | 0.16 |
| Data are presented as No./total (%) unless otherwise indicated. Column totals reflect the propensity-score–matched cohorts within each WBC stratum; denominators in individual rows may vary because of missing outcome data. Admission WBC strata were defined as <10 versus ≥10×10⁹/L.  **Primary endpoint.** The 90-day ordinal mRS (0–6) was analyzed using a proportional-odds model and is reported as the adjusted common odds ratio (aCOR), with values >1.00 favoring MPSS.  **Binary endpoints.** Multivariable logistic regression was used to estimate adjusted odds ratios (aORs). Where reported, adjusted risk differences (aRDs) were derived by marginal standardization.  **Continuous endpoints.** Linear regression was used to estimate adjusted mean differences (β).  **Covariates.** All models were adjusted for the MARVEL-aligned covariate set specified in the analysis plan: age, prestroke mRS score, baseline NIHSS score, baseline ASPECTS, use of intravenous thrombolysis, onset-to-randomization time, and occlusion site. To address residual imbalance after matching, the low-WBC stratum additionally adjusted for atrial fibrillation (SMD = 0.14); the high-WBC stratum additionally adjusted for diabetes (SMD = 0.11), ASITN/SIR collateral grade (SMD = 0.14), and TOAST subtype (SMD = 0.13).  **Multiplicity.** Holm–Bonferroni adjustment was applied separately within each WBC stratum to the family of four dichotomized functional thresholds: mRS 0–1, 0–2, 0–3, and 0–4. The two WBC strata were not pooled for this multiplicity adjustment. The primary ordinal endpoint, safety endpoints, and continuous endpoints were not multiplicity-adjusted. Reported table *P* values are nominal unless otherwise specified.  **Directionality.** For functional outcomes, aOR >1.00 or aRD >0 favors MPSS. For mortality and hemorrhagic outcomes, aOR <1.00 or aRD <0 favors MPSS. For NIHSS, lower values are better; for EQ-5D-VAS, higher values are better.  **Reporting.** Effect sizes are shown with 95% CIs; two-sided *P* values are reported.  **Abbreviations:** aCOR, adjusted common odds ratio; aOR, adjusted odds ratio; aRD, adjusted risk difference; ASITN/SIR, American Society of Interventional and Therapeutic Neuroradiology/Society of Interventional Radiology collateral grade; CI, confidence interval; EQ-5D-VAS, EuroQol 5-Dimension visual analog scale; IQR, interquartile range; MPSS, methylprednisolone sodium succinate; mRS, modified Rankin Scale; NIHSS, National Institutes of Health Stroke Scale; sICH, symptomatic intracranial hemorrhage; SMD, standardized mean difference; TOAST, Trial of Org 10172 in Acute Stroke Treatment; VAS, visual analog scale; WBC, white blood cell count. | | | | | | | | | |

## eTable 5. Clinical Outcomes by Treatment Within Admission WBC Strata: LASSO-Sensitivity Analyses

|  | | **No./total (%)** | | | | **No./total (%)** | | | |
| --- | --- | --- | --- | --- | --- | --- | --- | --- | --- |
|  | | **Low-WBC stratum (<10×10⁹/L, n=808)** | | | | **High-WBC stratum (≥10×10⁹/L, n=393)** | | | |
|  | | Placebo group (n=407) | MPSS group (n=401) | Adjusted value  (95% CI) | *P value* | Placebo group (n=190) | MPSS group (n=203) | Adjusted value  (95% CI) | *P value* |
| **Primary efficacy outcome** *a* | | |  |  |  |  |  |  |  |
| mRS score at 90 days, median (IQR) | | 3.0 (1.0–5.0) | 3.0 (1.0–5.0) | aCOR：1.01 (0.78-1.30) | 0.96 | 4.0 (2.0–6.0) | 3.0 (1.8–5.0) | aCOR：1.59 (1.11-2.33) | 0.01 |
| **Secondary efficacy outcomes** *b* | | |  |  |  |  |  |  |  |
|  | mRS score of 0-1 at 90 days | 125/404 (30.9) | 120/399 (30.1) | aOR: 0.92 (0.65–1.30) | 0.64 | 44/190 (23.2) | 50/202 (24.8) | aOR: 1.10 (0.65-1.85) | 0.72 |
|  | mRS score of 0-2 at 90 days | 190/404 (47.0) | 192/399 (47.9) | aOR: 1.09 (0.79–1.50) | 0.62 | 69/190 (26.3) | 91/202 (45.0) | aOR: 1.49 (0.94-2.33) | 0.09 |
|  | mRS score of 0-3 at 90 days | 249/404 (61.6) | 250/399 (62.7) | aOR: 1.10 (0.78–1.54) | 0.60 | 92/190 (45.8) | 124/202 (61.4) | aOR: 1.82 (1.15-2.86) | 0.01 |
|  | mRS score of 0-4 at 90 days | 290/404 (71.8) | 293/399 (73.4) | aOR: 1.20 (0.83–1.73) | 0.34 | 114/190 (60.0) | 146/202 (72.3) | aOR: 1.82 (1.14-2.94) | 0.01 |
|  | NIHSS score at 5–7 days, median (IQR) | 9.5 (3.0–19.0) | 10.0 (2.0–21.0) | β: 0.01 (-1.55–1.57) | 0.99 | 13 (6-31) | 11 (4.8-23) | β: -1.90 (-4.22, 0.43) | 0.11 |
|  | EQ-5D-VAS score at 90 days, median (IQR) | 60 (0–80) | 55 (5–85) | β: 0.27 (-4.02–4.56) | 0.90 | 30 (0-80) | 55 (5-80) | β: 7.58 (1.17, 13.99) | 0.02 |
| **Safety outcomes** | | |  |  |  |  |  |  |  |
|  | Mortality | 92/404 (22.8) | 85/399 (21.3) | aOR: 0.94 (0.57–1.24) | 0.38 | 63/190 (33.2) | 47/202 (23.3) | aOR: 0.57 (0.35-0.94) | 0.03 |
|  | Symptomatic intracranial hemorrhage | 35/403 (8.7) | 31/393 (7.9) | aOR: 0.90 (0.53–1.54) | 0.70 | 28/189 (14.8) | 25/202 (12.4) | aOR: 0.78 (0.42-1.45) | 0.43 |
|  | Any radiologic intracranial hemorrhage | 137/403 (34.0) | 133/393 (33.2) | aOR: 0.93 (0.69–1.26) | 0.64 | 81/189 (42.9) | 101/202 (50.0) | aOR: 1.35 (0.88-2.08) | 0.17 |
|  | Pneumonia | 197/407 (48.4) | 170/401 (42.4) | aOR: 0.78 (0.58–1.04) | 0.09 | 116/190 (61.1) | 101/202 (50.0) | aOR: 0.60 (0.39-0.93) | 0.02 |
|  | Gastrointestinal bleeding within 7 days after EVT | 21/407 (5.2) | 12/401 (3.0) | aOR: 0.39 (0.18–0.87) | 0.02 | 18/190 (9.5) | 13/203 (6.4) | aOR: 0.64 (0.30-1.37) | 0.25 |
| Data are presented as No./total (%) unless otherwise indicated. Column totals reflect the baseline-covariate complete-case cohort used for the LASSO-sensitivity analyses; denominators in individual rows may vary because of missing outcome data. Admission WBC strata were defined as <10 versus ≥10×10⁹/L.  **Primary endpoint.** The 90-day ordinal mRS (0–6) was analyzed using a proportional-odds model and is reported as the adjusted common odds ratio (aCOR), with values >1.00 favoring MPSS.  **Binary endpoints.** Multivariable logistic regression was used to estimate adjusted odds ratios (aORs). Where reported, adjusted risk differences (aRDs) were derived by marginal standardization.  **Continuous endpoints.** Linear regression was used to estimate adjusted mean differences (β).  **Covariates.** Models used LASSO-selected covariate sets as described in eMethods 3. In the low-WBC stratum, models adjusted for age, hypertension, hyperlipidemia, lymphocyte count, glucose, baseline NIHSS score, baseline ASPECTS, ASITN/SIR collateral grade, TOAST subtype, occlusion site, anesthesia type, onset-to-puncture time, and puncture-to-reperfusion time. In the high-WBC stratum, models adjusted for age, baseline NIHSS score, baseline ASPECTS, ASITN/SIR collateral grade, and puncture-to-reperfusion time.  **Multiplicity.** Holm–Bonferroni adjustment was applied separately within each WBC stratum to the family of four dichotomized functional thresholds: mRS 0–1, 0–2, 0–3, and 0–4. The two WBC strata were not pooled for this multiplicity adjustment. The primary ordinal endpoint, safety endpoints, and continuous endpoints were not multiplicity-adjusted. Reported table *P* values are nominal unless otherwise specified.  **Directionality.** For functional outcomes, aOR >1.00 or aRD >0 favors MPSS. For mortality and hemorrhagic outcomes, aOR <1.00 or aRD <0 favors MPSS. For NIHSS, lower values are better; for EQ-5D-VAS, higher values are better.  **Reporting.** Effect sizes are shown with 95% CIs; two-sided *P* values are reported.  **Abbreviations:** aCOR, adjusted common odds ratio; aOR, adjusted odds ratio; aRD, adjusted risk difference; ASITN/SIR, American Society of Interventional and Therapeutic Neuroradiology/Society of Interventional Radiology collateral grade; CI, confidence interval; EQ-5D-VAS, EuroQol 5-Dimension visual analog scale; IQR, interquartile range; LC, lymphocyte count; MPSS, methylprednisolone sodium succinate; mRS, modified Rankin Scale; NIHSS, National Institutes of Health Stroke Scale; sICH, symptomatic intracranial hemorrhage; TOAST, Trial of Org 10172 in Acute Stroke Treatment; WBC, white blood cell count. | | | | | | | | | |

## eTable 6. Clinical Outcomes by Treatment Within Admission WBC Strata: Multiple-Imputation Sensitivity Analyses

|  | | **No./total (%)** | | | | **No./total (%)** | | | |
| --- | --- | --- | --- | --- | --- | --- | --- | --- | --- |
|  | | **Low-WBC stratum (<10×10⁹/L)** | | | | **High-WBC stratum (≥10×10⁹/L)** | | | |
|  | | Placebo group | MPSS group | Adjusted value  (95% CI) | *P value* | Placebo group | MPSS group | Adjusted value  (95% CI) | *P value* |
| **Primary efficacy outcome** | | |  |  |  |  |  |  |  |
| mRS score at 90 days, median (IQR) | | 3(1–5) | 3 (1–5) | aCOR: 0.98 (0.78 to 1.23) | 0.85 | 4 (2–6) | 3 (1–5) | aCOR: 1.53 (1.09 to 2.15) | **0.01** |
| **Secondary efficacy outcomes** | | |  |  |  |  |  |  |  |
|  | mRS score of 0-1 at 90 days | 154.8/502 | 146.2/491.8 | aOR: 0.90 (0.66 to 1.22) | 0.50 | 59.2/247 | 65.8/259.2 | aOR: 1.10 (0.69 to 1.76) | 0.69 |
|  | mRS score of 0-2 at 90 days | 239.4/502 | 237.4/491.8 | aOR: 0.99 (0.75 to 1.33) | 0.98 | 92.6/247 | 117.6/259.2 | aOR: 1.45 (0.95 to 2.24) | 0.09 |
|  | mRS score of 0-3 at 90 days | 313/502 | 314.2/491.8 | aOR: 1.03 (0.76 to 1.40) | 0.86 | 127/247 | 158.8/259.2 | aOR: 1.60 (1.03 to 2.50) | **0.04** |
|  | mRS score of 0-4 at 90 days | 362.6/502 | 369/491.8 | aOR: 1.15 (0.84 to 1.59) | 0.38 | 155.4/247 | 189/259.2 | aOR: 1.68 (1.10 to 2.56) | **0.02** |
|  | NIHSS score at 5–7 days, median (IQR) | 9 (3-19) | 9 (2, 19) | β: -0.19 (-1.69 to 1.30) | 0.80 | 13 (7-32) | 11 (5-23) | β: -1.83 (-3.99 to 0.34) | 0.10 |
|  | EQ-5D-VAS score at 90 days, median (IQR) | 60 (0-80) | 65 (15-85) | β: 0.28 (-3.84 to 4.39) | 0.90 | 30 (0-80) | 55 (5-80) | β: 6.68 (0.45 to 12.91) | 0.04 |
| **Safety outcomes** | | |  |  |  |  |  |  |  |
|  | Mortality | 114.4/502 | 98.8/491.8 | aOR: 0.86 (0.62 to 1.21) | 0.40 | 77.6/247 | 57.2/259.2 | aOR: 0.59 (0.38 to 0.91) | 0.02 |
|  | Symptomatic intracranial hemorrhage | 43/501 | 35/485 | aOR: 0.85 (0.52 to 1.37) | 0.50 | 37/244 | 30/259 | aOR: 0.75 (0.43 to 1.31) | 0.31 |
|  | Any radiologic intracranial hemorrhage | 162.6/501 | 160/485 | aOR: 1.04 (0.78 to 1.37) | 0.80 | 102.4/244 | 124/259 | aOR: 1.35 (0.92 to 1.97) | 0.16 |
|  | Pneumonia | 250.8/505 | 207.4/493.8 | aOR: 0.75 (0.57 to 0.99) | 0.04 | 153.2/247 | 127.6/260.2 | aOR: 0.57 (0.38 to 0.85) | 0.01 |
|  | Gastrointestinal bleeding within 7 days after EVT | 23.2/505 | 13.6/493.8 | aOR: 0.56 (0.23 to 1.14) | 0.11 | 19.8/247 | 15.4/260.2 | aOR: 0.70 (0.34 to 1.44) | 0.33 |
| Data are presented as No./total (%) unless otherwise indicated. Values for binary outcomes represent pooled event counts and denominators averaged across imputed datasets; therefore, non-integer counts may appear. Admission WBC strata were defined as <10 versus ≥10×10⁹/L. Missing baseline covariates were imputed using multiple imputation by chained equations; admission WBC was required for stratification and was not imputed. Outcomes were analyzed as observed and were not imputed.  **Primary endpoint.** The 90-day ordinal mRS (0–6) was analyzed using a proportional-odds model and is reported as the adjusted common odds ratio (aCOR), with values >1.00 favoring MPSS.  **Binary endpoints.** Multivariable logistic regression was used to estimate adjusted odds ratios (aORs). Where reported, adjusted risk differences (aRDs) were derived by marginal standardization.  **Continuous endpoints.** Linear regression was used to estimate adjusted mean differences (β).  **Covariates.** Models adjusted for the MARVEL-aligned covariate set specified in the analysis plan: age, prestroke mRS score, baseline NIHSS score, baseline ASPECTS, use of intravenous thrombolysis, onset-to-randomization time, and occlusion site.  **Multiplicity.** Holm–Bonferroni adjustment was applied separately within each WBC stratum to the family of four dichotomized functional thresholds: mRS 0–1, 0–2, 0–3, and 0–4. The two WBC strata were not pooled for this multiplicity adjustment. The primary ordinal endpoint, safety endpoints, and continuous endpoints were not multiplicity-adjusted. Reported table *P* values are nominal unless otherwise specified.  **Directionality.** For functional outcomes, aOR >1.00 or aRD >0 favors MPSS. For mortality and hemorrhagic outcomes, aOR <1.00 or aRD <0 favors MPSS. For NIHSS, lower values are better; for EQ-5D-VAS, higher values are better.  **Reporting.** Effect sizes are shown with 95% CIs; two-sided *P* values are reported. Estimates from imputed datasets were combined using Rubin’s rules.  **Abbreviations:** aCOR, adjusted common odds ratio; aOR, adjusted odds ratio; aRD, adjusted risk difference; CI, confidence interval; EQ-5D-VAS, EuroQol 5-Dimension visual analog scale; IQR, interquartile range; MI, multiple imputation; MPSS, methylprednisolone sodium succinate; mRS, modified Rankin Scale; NIHSS, National Institutes of Health Stroke Scale; sICH, symptomatic intracranial hemorrhage; VAS, visual analog scale; WBC, white blood cell count. | | | | | | | | | |

# Figures

## eFigure S1. Love Plots of Standardized Mean Differences Before and After Matching, by WBC Stratum

**Panel A. WBC <10×10⁹/L stratum.**
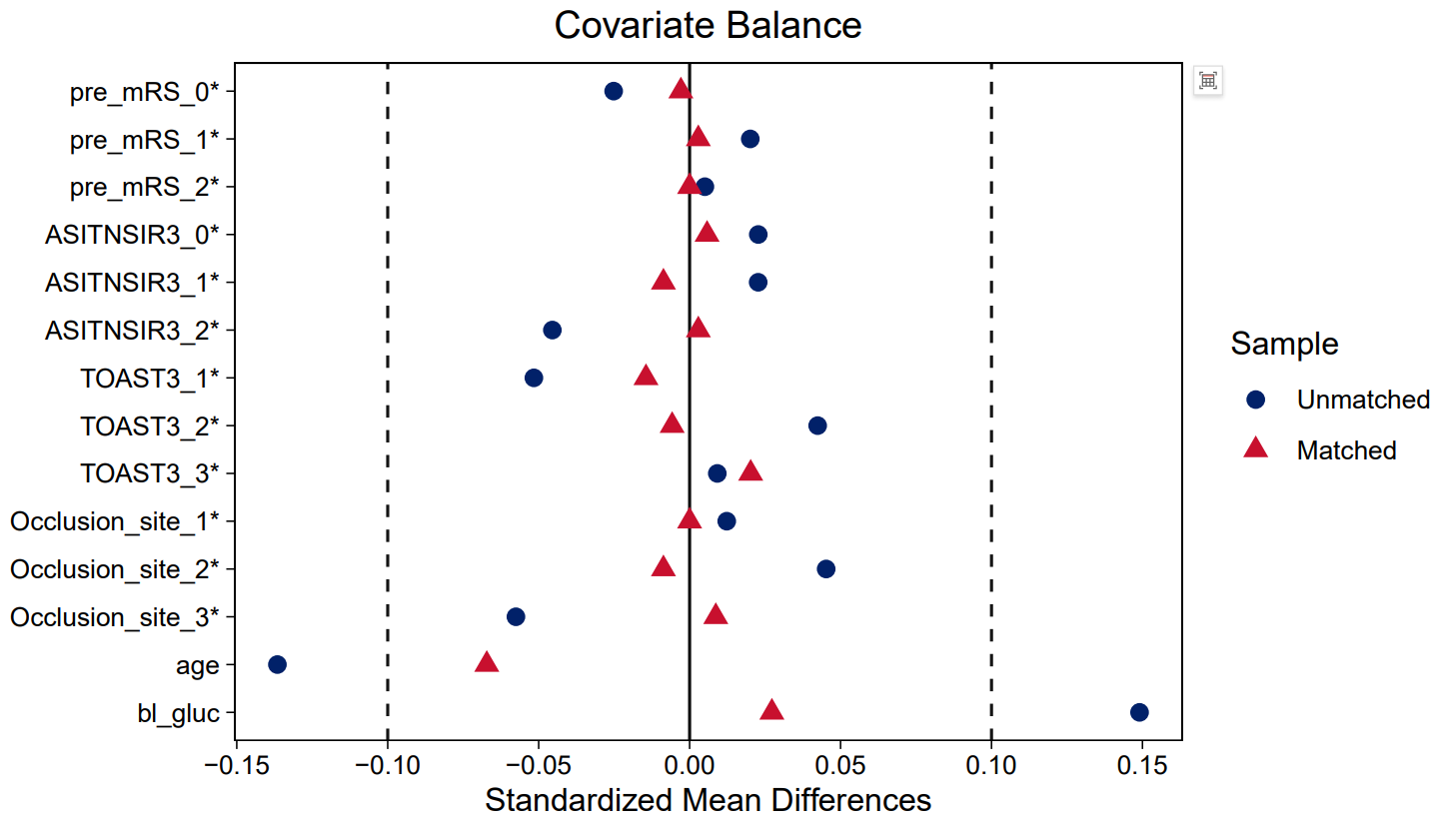


**Panel B. WBC ≥10×10⁹/L stratum.**


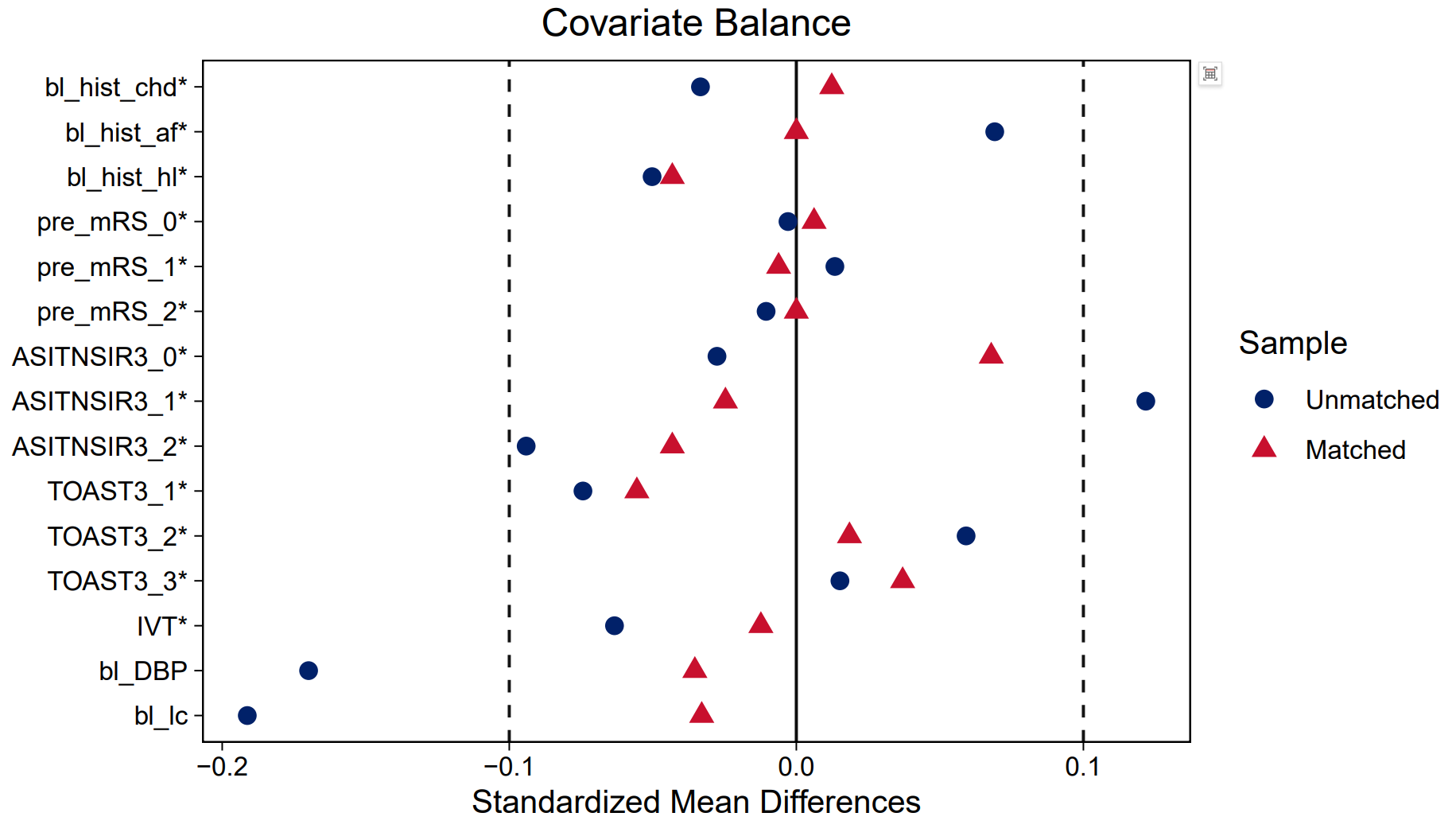


**Caption.** Propensity scores were estimated from the prespecified baseline covariates (see eMethods 3) and patients were matched 1:1 on the logit of the propensity score with a caliper of 0.10, without replacement. Love plots display standardized mean differences for each covariate before and after matching, with |SMD|=0.10 as the balance criterion. Post-matching, most covariates achieved |SMD|<0.10; minor residual imbalances are detailed in eTable 3.

## eFigure S2. LASSO Variable Selection: Cross-Validation Error Curves and Coefficient Paths

**Panel A.** Overall cohort (interaction models).


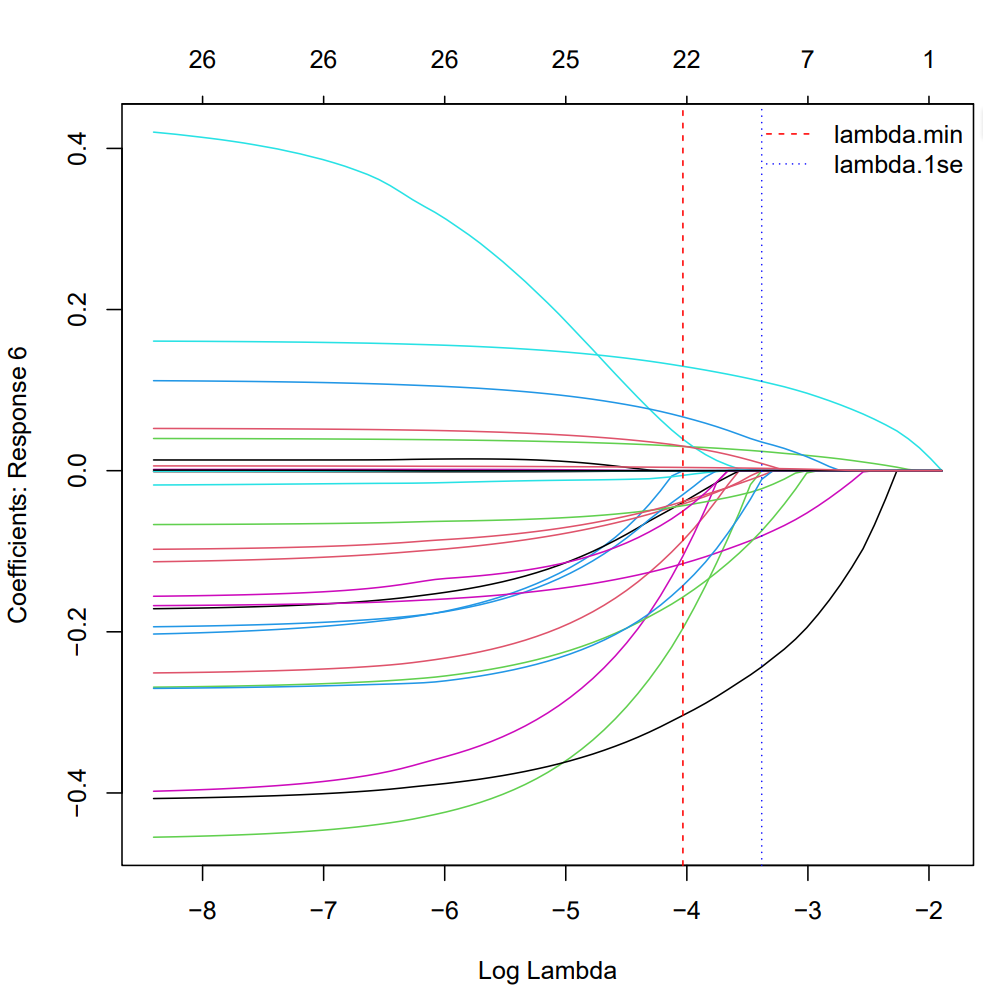

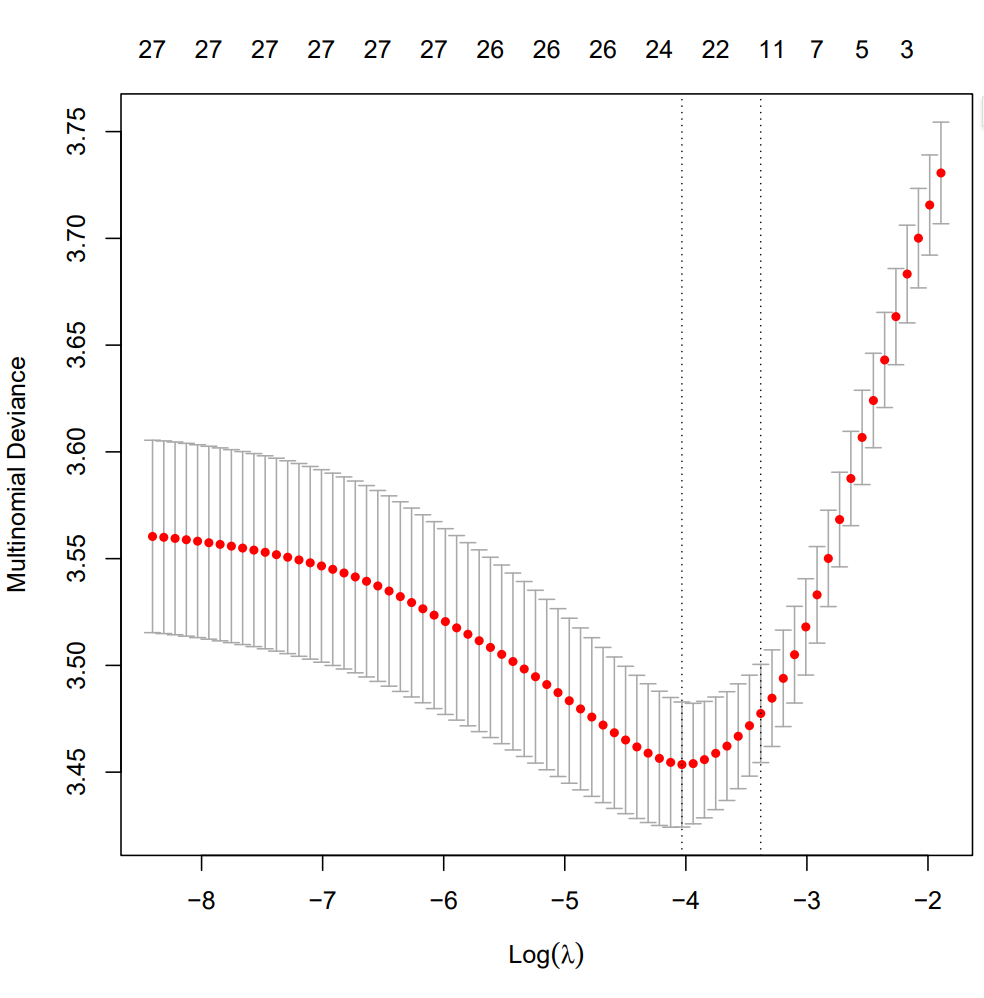


**Panel B.** WBC <10×10⁹/L stratum.


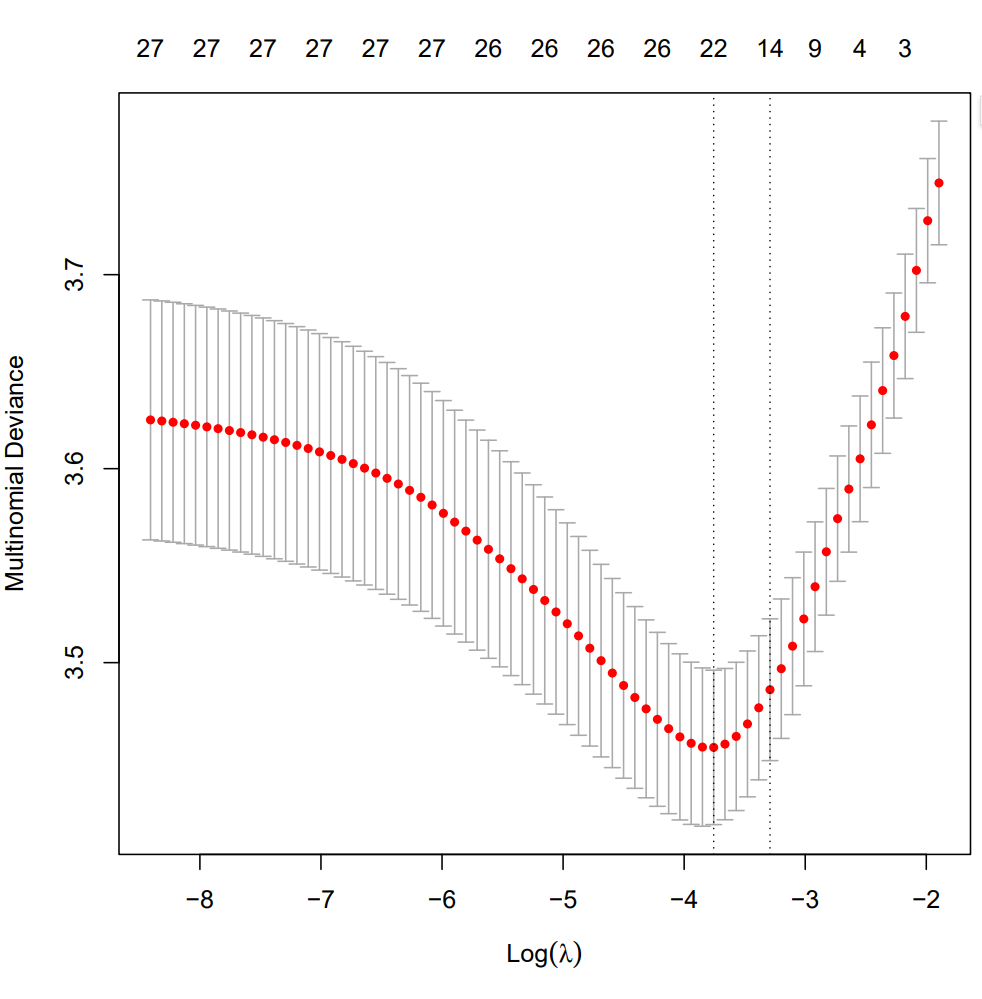

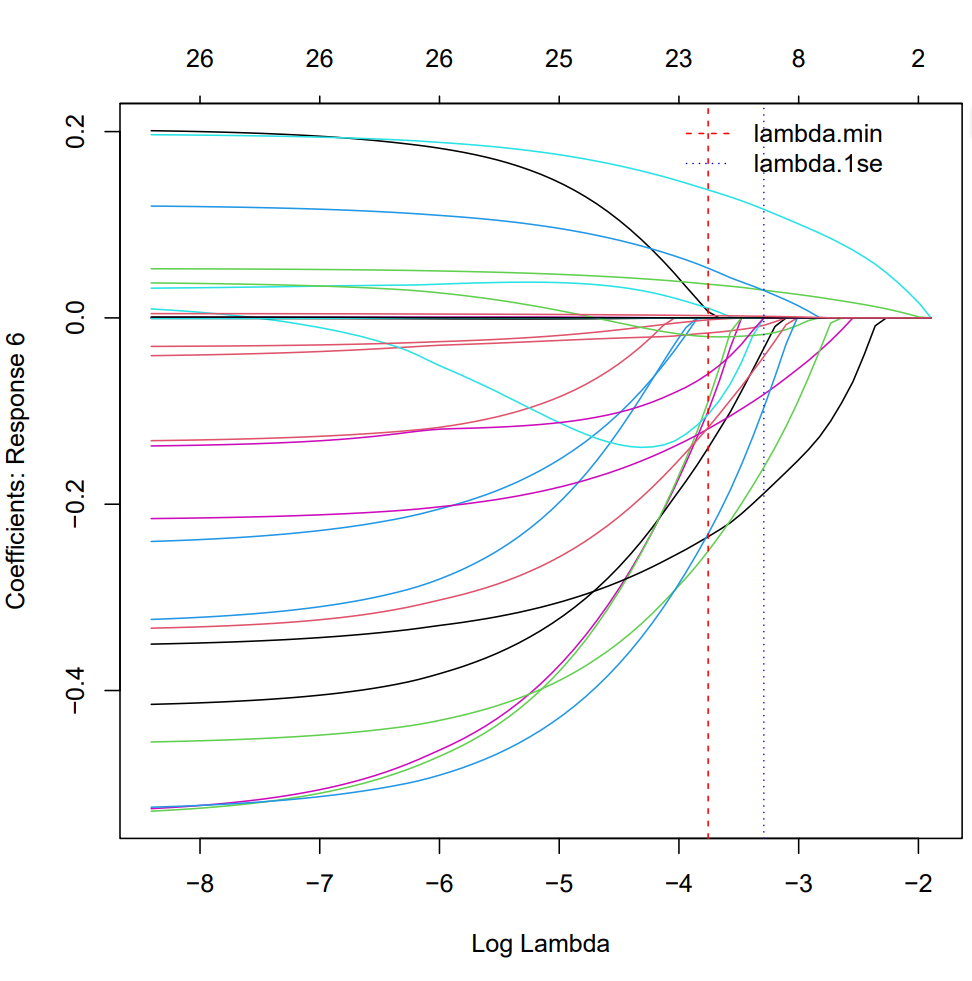


**Panel C.** WBC ≥10×10⁹/L stratum.


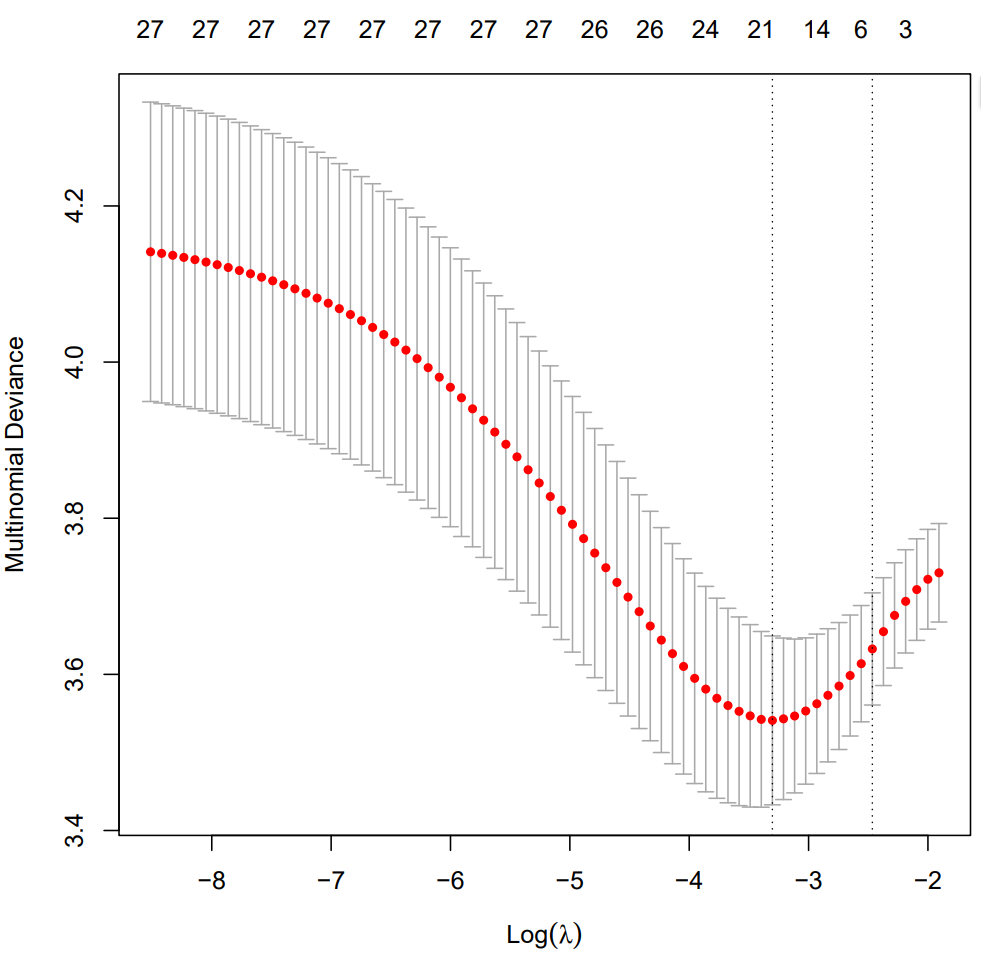

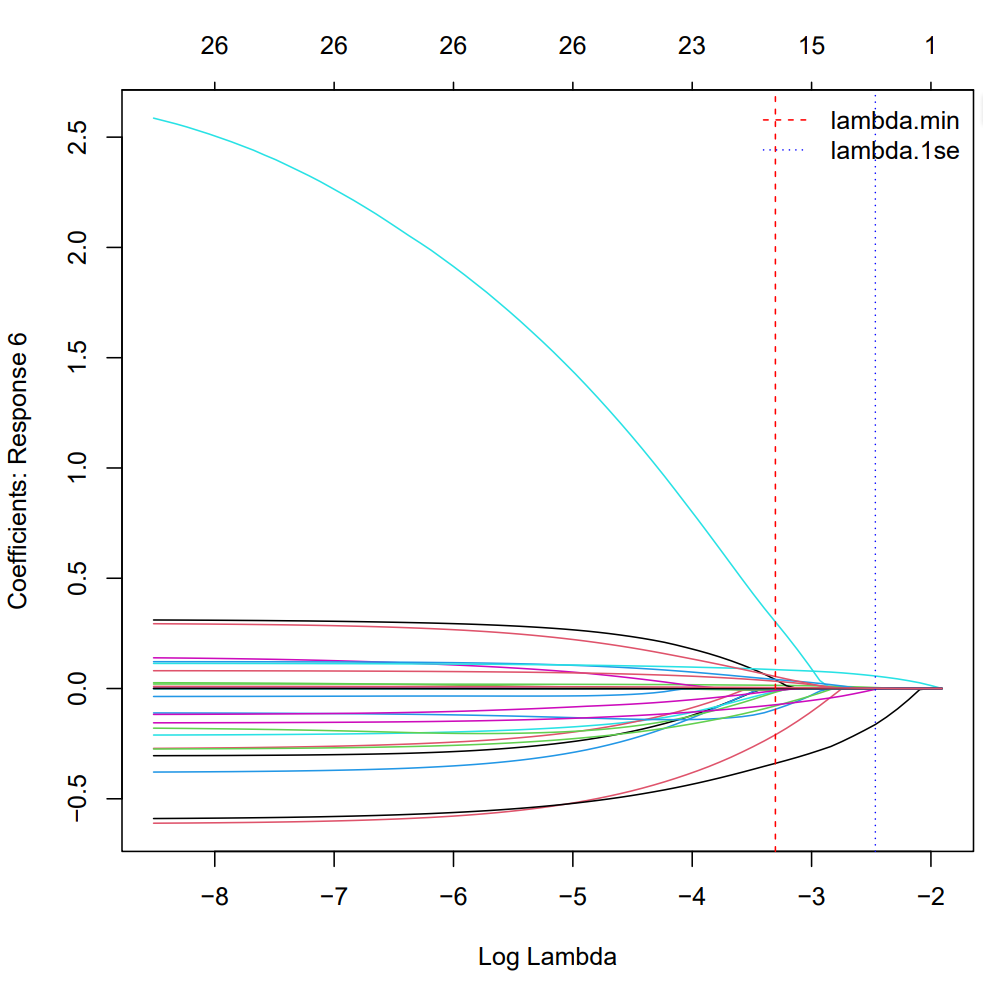


**Caption.** LASSO was fit with 10-fold cross-validation; λ.1se was used to define the parsimonious model in each analysis set. Plots show the mean CV error (with SE bars) across λ values and the regularization paths of standardized coefficients.

## eFigure S3 Horizontal Dot Plot of Missing Observations per Variable.


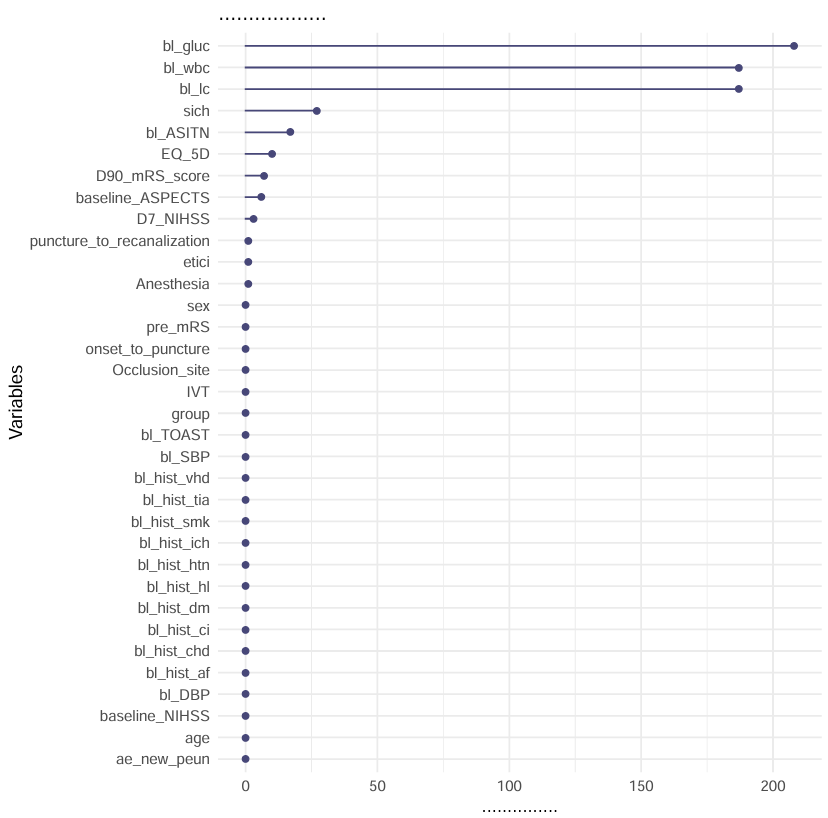


Caption. Counts of missing observations for each variable (ordered by decreasing missingness). Missingness is concentrated in baseline laboratory indices (e.g., glucose, WBC, lymphocyte count). All variables, together with treatment and outcome, entered the MICE under a Missing-At-Random assumption.

## eFigure S4. Multiple-Imputation Diagnostics (MICE)

**Panel A. Convergence and chain-mixing: trace plots of the mean and SD across imputations for WBC, NIHSS, and ASPECTS over 20 iterations show stable mixing without drift, supporting convergence.**
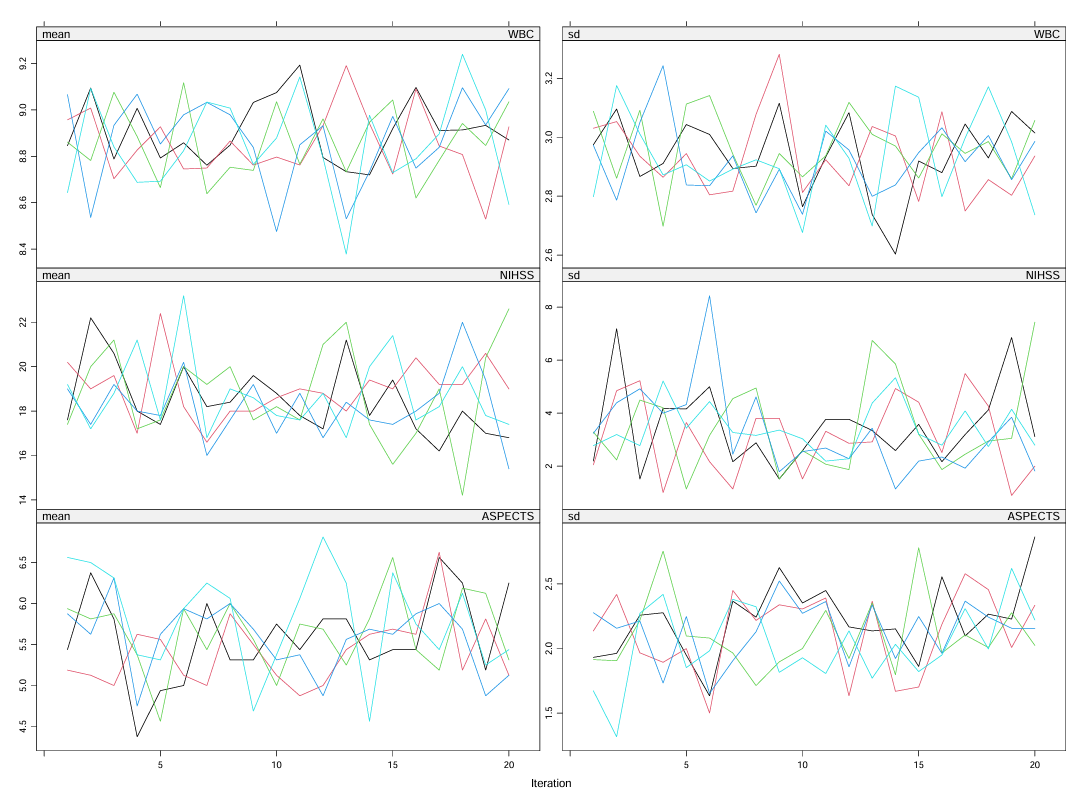


**Panel B. Strip plots by imputation index (1 = original dataset; 2–6 = imputed datasets), with blue = observed and red/pink = imputed. Distributions are comparable across imputations.**
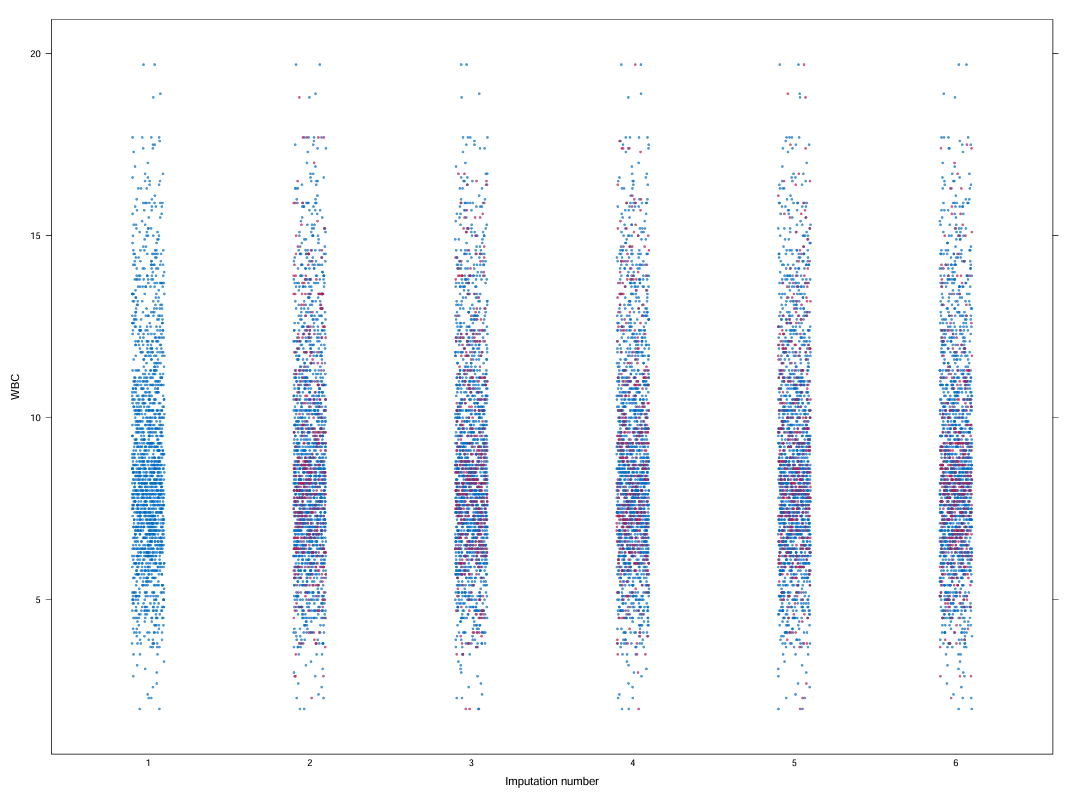


**Panel C. Kernel-density overlays (blue = observed; red/pink = imputed) for WBC, NIHSS, ASPECTS, and glucose. Observed and imputed distributions align in central tendency and shape, supporting plausibility under MAR.**
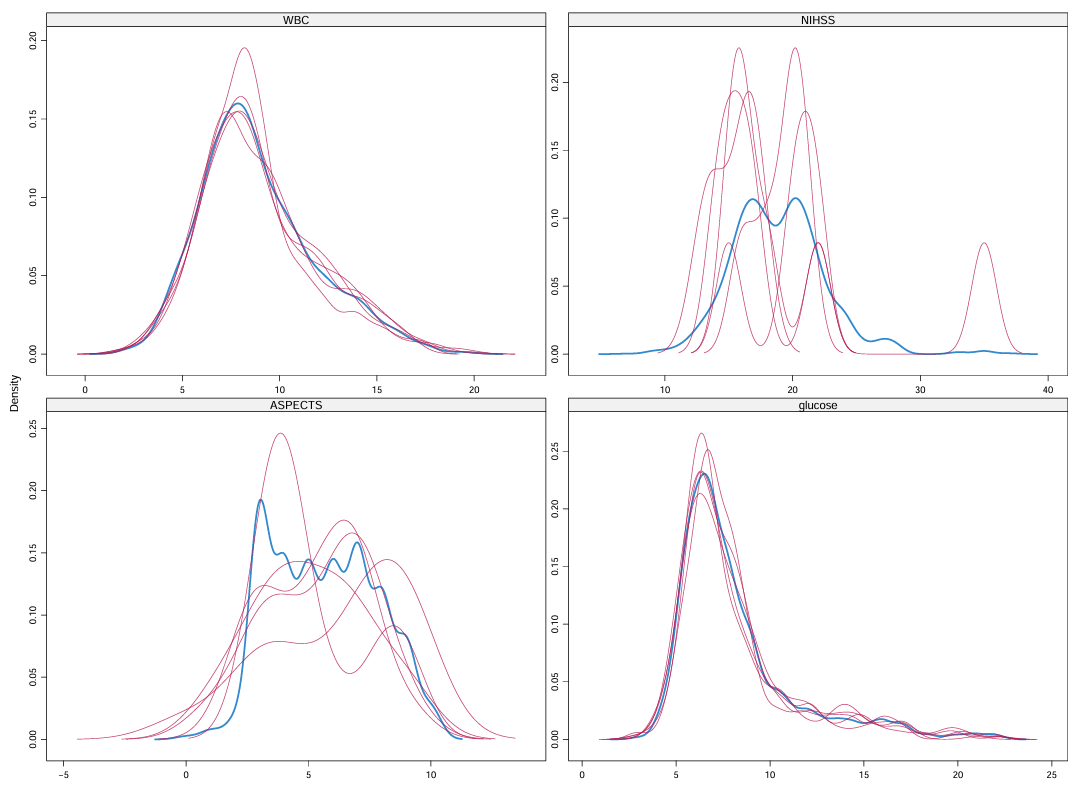


Caption. Diagnostics for MICE with m=5 imputations and 20 iterations.

## eFigure S5. Sankey Diagram of Patient Flows by Baseline WBC Stratum, Treatment, and 90-Day mRS


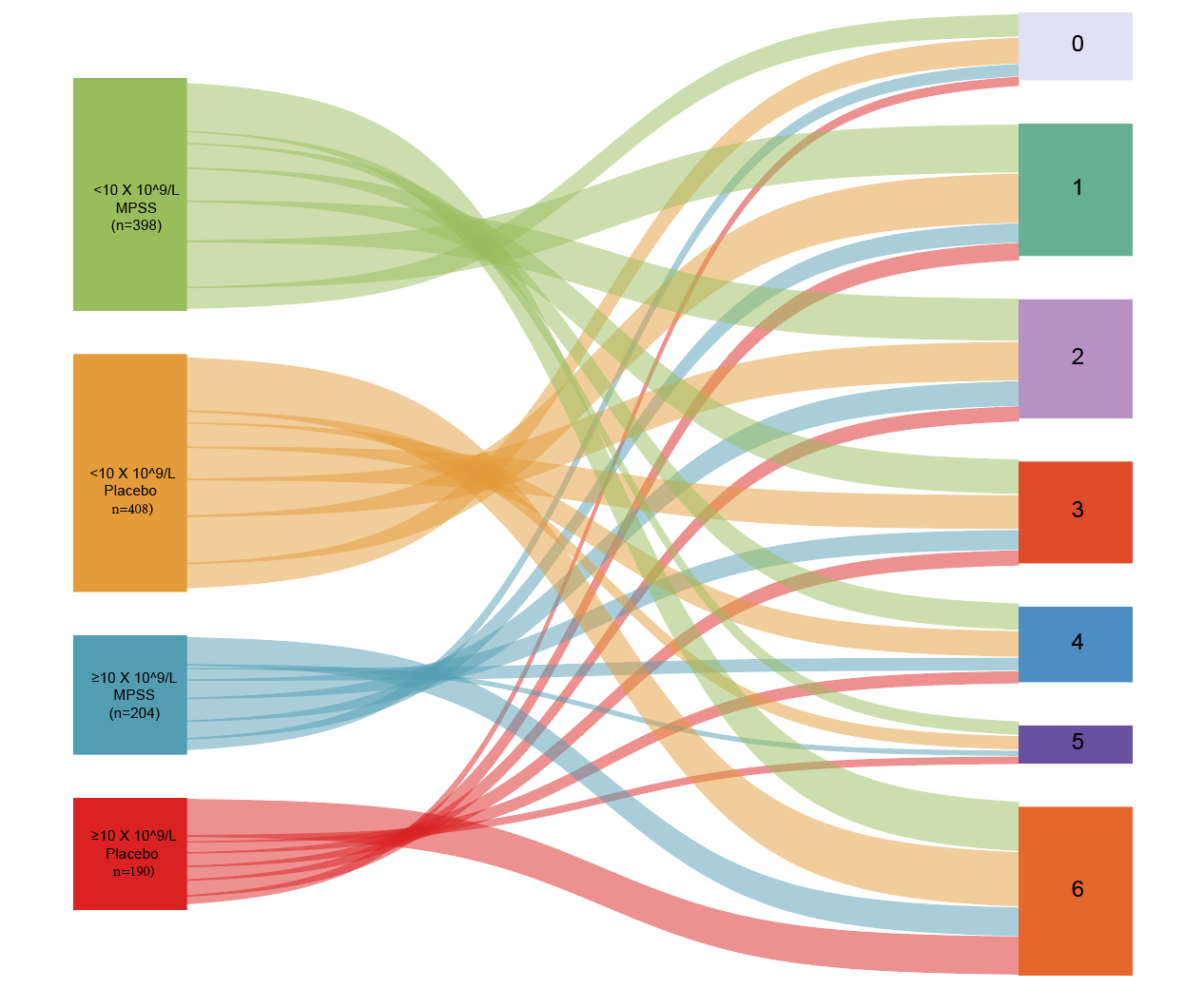


**Caption.** The four left nodes represent the stratum–treatment combinations: WBC <10×10⁹/L with MPSS, WBC <10×10⁹/L with Placebo, WBC ≥10×10⁹/L with MPSS, and WBC ≥10×10⁹/L with Placebo. Ribbon widths are proportional to the number of patients (percent) following each path; ribbon color encodes the source node. Flows from the WBC ≥10×10⁹/L + MPSS node visually tend to terminate in lower mRS categories compared with WBC ≥10×10⁹/L + Placebo, whereas differences are small within WBC <10×10⁹/L.
